# The scRNA-seq Expression Profiling of the Receptor ACE2 and the Cellular Protease TMPRSS2 Reveals Human Organs Susceptible to SARS-CoV-2 Infection

**DOI:** 10.3390/ijerph18010284

**Published:** 2021-01-02

**Authors:** Jing Qi, Yang Zhou, Jiao Hua, Liying Zhang, Jialin Bian, Beibei Liu, Zicen Zhao, Shuilin Jin

**Affiliations:** School of Mathematics, Harbin Institute of Technology, Harbin 150001, China; 19b312002@stu.hit.edu.cn (J.Q.); 19s012001@stu.hit.edu.cn (Y.Z.); hjhuajiao@163.com (J.H.); 18s112063@stu.hit.edu.cn (L.Z.); jialinbian@163.com (J.B.); 18845722421@163.com (B.L.); 3544144971@nefu.edu.cn (Z.Z.)

**Keywords:** SARS-CoV-2, COVID-19, scRNA-seq, susceptible organs, ACE2, TMPRSS2

## Abstract

COVID-19 patients always develop multiple organ dysfunction syndromes other than lungs, suggesting the novel virus SARS-CoV-2 also invades other organs. Therefore, studying the viral susceptibility of other organs is important for a deeper understanding of viral pathogenesis. Angiotensin-converting enzyme II (ACE2) is the receptor protein of SARS-CoV-2, and TMPRSS2 promotes virus proliferation and transmission. We investigated the ACE2 and TMPRSS2 expression levels of cell types from 31 organs to evaluate the risk of viral infection using single-cell RNA sequencing (scRNA-seq) data. For the first time, we found that the gall bladder and fallopian tube are vulnerable to SARS-CoV-2 infection. Besides, the nose, heart, small intestine, large intestine, esophagus, brain, testis, and kidney are also identified to be high-risk organs with high expression levels of ACE2 and TMPRSS2. Moreover, the susceptible organs are grouped into three risk levels based on the ACE2 and TMPRSS2 expression. As a result, the respiratory system, digestive system, and urinary system are at the top-risk level for SARS-CoV-2 infection. This study provides evidence for SARS-CoV-2 infection in the human nervous system, digestive system, reproductive system, respiratory system, circulatory system, and urinary system using scRNA-seq data, which helps in the clinical diagnosis and treatment of patients.

## 1. Introduction

In December 2019, a novel coronavirus pneumonia recently named Coronavirus Disease 2019 (COVID-19) by the World Health Organization (WHO) [1], was first reported in several patients in Wuhan, China [2,3,4,5]. As of December 2020, COVID-19 has spread widely in more than 200 countries; more than 71.43 million people have suffered from the disease, and over 1.6 million people have died, posing a major threat to global public health.

COVID-19 is caused by severe acute respiratory syndrome coronavirus 2 (SARS-CoV-2) [1,6,7], which seriously damages the respiratory system. Patients develop acute respiratory infection symptoms, and even acute respiratory distress syndrome (ARDS), acute respiratory failure, and other severe symptoms [4,5,8,9]. On the other side, complications that occurred outside the lungs, including acute kidney injury, liver function damage, and multiple organ failure, were commonly described in COVID-19 cases and even led to death, suggesting that the virus invades other organs at the same time [4,5,10,11,12,13]. SARS-CoV-2 enters the cell via the binding of spike (S) protein and angiotensin-converting enzyme II (ACE2) [14,15,16,17], the receptor protein of SARS-CoV, and NL63 [18,19,20,21]. The anti-ACE2 antibody blocks the viral entry driven by SARS-CoV-2 S protein [17]. In addition, human adenocarcinoma cells (A549) were found to be incompatible with SARS-CoV-2 infection because of the low expression of ACE2 [22,23]. The cellular protease TMPRSS2 primes the S protein and promotes the transmission during the viral infection, and the inhibitor of TMPRSS2 blocks the SARS-CoV-2 infection of lung cells [17,24]. Thus, the expression of ACE2 and TMPRSS2 is indispensable for viral infection in cells. The distribution and expression of ACE2 and TMPRSS2 are strongly associated with the target organ of the SARS-CoV-2 infection [25,26,27,28]. Previous studies have shown that smoking and aging induce an increase in ACE2 expression in the human respiratory tissue, while smokers and people with old age are vulnerable to COVID-19 [25,29,30,31]. It has been shown that the androgens can up-regulate the expression of ACE2 and TMPRSS2, which is related to the higher mortality and morbidity of males relative to females in the COVID-19 pandemic [31,32]. Moreover, several studies suggested that the blocking of androgen signaling and the down-regulation of ACE2 and TMPRSS2 are protective against COVID-19 [26,33]. Targeting the transcriptional regulation of TMPRSS2 and ACE2 has been an important strategy to prevent SARS-CoV-2 infection in clinical treatment [32,34].

Single-cell RNA sequencing (scRNA-seq) can obtain the gene expression profile of a single cell, which better reveals the heterogeneity of cells, and allows better understanding of the functions of an individual cell in its microenvironment [35,36,37,38,39]. Therefore, scRNA-seq technology provides a tool to study the pathogenic mechanism of SARS-CoV-2 on human cells from the expression of genes in cell resolution. The pathological inference and gross anatomical observations indicated that the lesions caused by the novel coronavirus were mainly in the lung [9,40,41]. ACE2 is mainly expressed in type II alveolar cells (AT2) in the lung [42,43,44], which implies that the AT2 cell is vulnerable to SARS-CoV-2 infection. Researchers have obtained the susceptibility of other organs from different systems using ACE2 expression in AT2 cells as a baseline. Following this way, many respiratory districts were considered, and ACE2 was reported to be highly expressed in the nasal tissue, mouth, airway, and lung [43,45,46]. The esophagus, large intestine (ileum and colon), and pancreas were identified as high-risk organs in the digestive system [43,46,47,48]. The kidney and bladder, as major organs of the urinary system, were also indicated to be high ACE2-expressed [43,49,50,51]. Besides, the testes and uterus were manifested to be susceptible organs, implying that the reproductive system was a potential route of viral infection [52,53]. Several studies have utilized the expression of ACE2 and TMPRSS2 to predict organ susceptibility. Zhou et al. analyzed human post-mortem eyes and surgical specimens for the expression of ACE2 and TMPRSS2, and results show that the ocular surface cells including conjunctiva are at high risk for SARS-CoV-2 infection [54]. Seow et al. reported the co-expression of ACE2 and TMPRSS2 in a TROP2+ liver progenitor population and identified a potentially high-risk liver cell-type for viral ingress [55]. Lukassen et al. investigated the expression levels and distributions of ACE2 and TMPRSS2 across cell types in lung tissue and bronchial branches, respectively [56]. Furthermore, ACE2 and TMPRSS2 are highly expressed in a transient secretory cell type of bronchial branches, suggesting the increased vulnerability for SARS-CoV-2 infection in this cell type [56].

In this paper, the expression level of ACE2 and TMPRSS2, in different cell types of organs from nine major systems (including the respiratory system, digestive system, nervous system, endocrine system, reproductive system, circulatory system, urinary system, and motor system), were obtained using the scRNA-seq data. For the first time, we found that the fallopian tube and gall bladder are vulnerable to SARS-CoV-2 infection. Besides, the nose (nasal brushing epithelial cells, nasal turbinate epithelial cells, and nasal airway epithelial cells), heart, small intestine (jejunum, ileum, and duodenum), large intestine (rectum and colon), esophagus, brain (substantia nigra and cortex), testes, and kidney are predicted as high-risk organs under a more rigorous standard. Moreover, as the spike (S) protein initiated by TMPRSS2 is essential for the entry of the virus into the target cells and the transmission of the virus in the infected host, we combined the expression level of ACE2 with the expression level of TMPRSS2 to predict the risk level of each susceptible organ, and found that the respiratory system, digestive system, and urinary system are at the highest level of vulnerability to SARS-CoV-2 infection.

## 2. Materials and Methods

The available scRNA-seq data of healthy humans were collected for the analysis, including 31 organs from nine major human systems (Table 1). The data were downloaded from the Gene Expression Omnibus (GEO) database and the Tissue Stability Cell Atlas, and the details of the data resources are in Supplementary File Table S1.

In our paper, analysis of scRNA-seq data was conducted in R environment (version 3.6.1; The R Foundation for Statistical Computing, Topeka, KS, USA) using Seurat package (https://github.com/satijalab/seurat). First, according to the number of expressed genes, counts and cells with mitochondrial content, the low-quality cells were filtered, and the cells were reserved within the range of μ−σ and μ+σ (where μ is the mean and σ is the standard deviation of the numbers). Then, the data were normalized by the logarithmic transformation and the downstream analysis was carried out with the top 2000 most variable genes. The ScaleData function were used for linear regression and principal component analysis (PCA) was performed on the scaled data for linear dimensional reduction. Finally, we performed the cell cluster analysis, gene differential expression analysis by t-test, and annotated the cell subtypes.

Here, the gene-expressed cell means that the gene expression value UMI (unique molecular identifier) is greater than or equal to 1, or the TPM (transcripts per million) is greater than 0, or the FPKM (fragments per kilobase million) is greater than 0. The ratio of ACE2-expressed cells and the ratio of TMPRSS2-expressed cells of the cell cluster are denoted as $R_{ACE2}$ and $R_{TMPRSS2}$, respectively. The average $R_{ACE2}$ over total AT2 cells across 8 samples is 0.79%. If $R_{ACE2}\geq 0.79\%$ and $R_{TMPRSS2}>0$, the cells in the cluster are identified as highly susceptible cells, and the corresponding organs are inferred as vulnerable to COVID-19. Moreover, we used the geometric mean of the ratios of TMPRSS2-expressed cells and the ratios of ACE2-expressed cells as the risk parameter to predict the risk for SARS-CoV-2 infection, and the risk parameter is denoted as:

(1) $$R=\sqrt{R_{ACE2}\cdot R_{TMPRSS2}}$$

According to the risk parameters, the susceptible organs were sorted into three groups. Specifically, the cells with a risk parameter greater than 0.1 are defined as level 1, which is the highest level of risk; the cells with a risk parameter of more than 0.05 and less than 0.1 are level 2, which denotes a higher risk; the rest are level 3, meaning there is an existing risk of infection.

## 3. Results

### 3.1. Respiratory System

**Figure 1:**
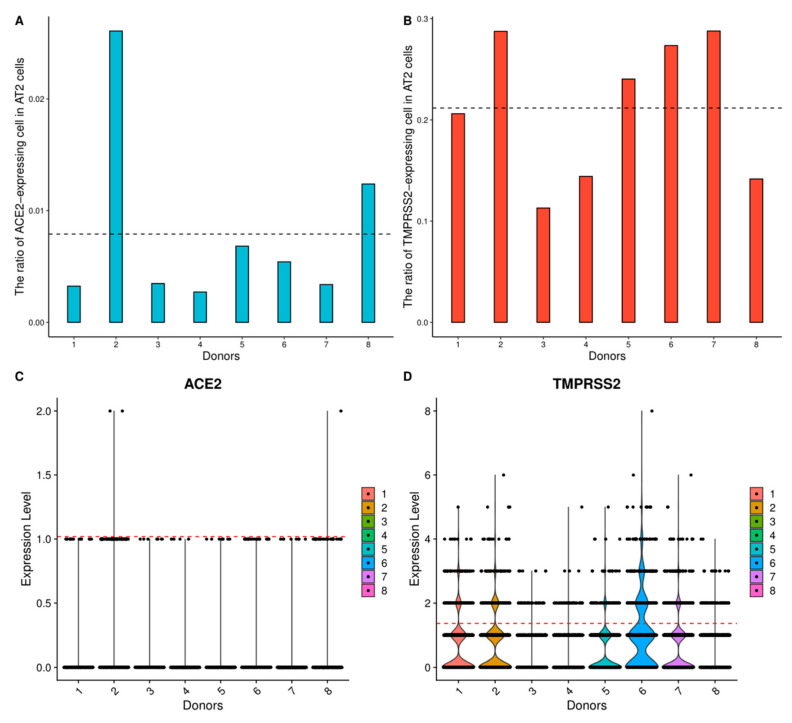
High angiotensin-converting enzyme II (ACE2) and transmembrane serine protease 2 (TMPRSS2) expression levels of type II alveolar (AT2) cells in the lung. (**A**), (**B**) $R_{ACE2}$ and $R_{TMPRSS2}$ in AT2 cells of 8 samples, respectively. The black dotted lines represent the corresponding average ratios across 8 samples. (**C**), (**D**) The expression distribution of ACE2 and TMPRSS2 in AT2 cells across 8 samples, respectively. The red dotted lines represent the corresponding average expression values of gene-expressed cells across 8 samples.

The scRNA-seq data of the lung, nose, trachea, and bronchus in the respiratory system were collected for analysis. In the lung, AT2 cells contain an average of 0.79% ACE2-expressed cells and 21.20% TMPRSS2-expressed cells across eight samples (Figure 1A,B, Supplementary File: Figures S1–S8, and Table 2), and the expression levels of ACE2 and TMPRSS2 are high in AT2 cells (Figure 1C,D). The data of nose (nasal brushing epithelial cells, nasal turbinate epithelial cells, and nasal airway epithelial cells) contain ACE2-expressed and TMPRSS2-expressed cell clusters (Supplementary File: Figure S9–S11), and the ratios of ACE2-expressed cells of these cell clusters are all above 0.79% (Table 2); thus, the nose is identified as the high-risk organ. The low ratio of ACE2-expressed cells in the bronchus and trachea means that they are low-risk infection organs (Supplementary File: Figures S12–S13).

### 3.2. Digestive System

**Figure 2:**
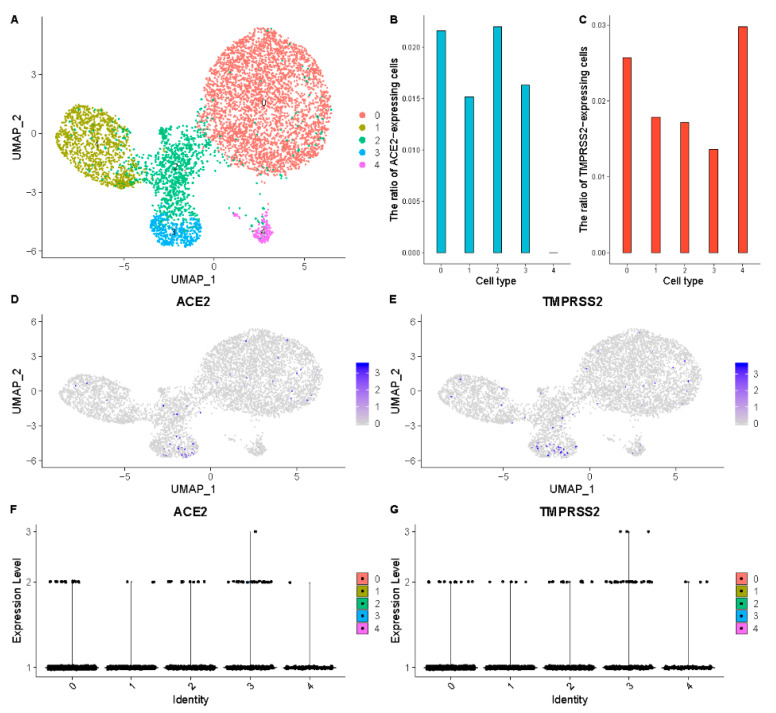
High ACE2 and TMPRSS2 expression levels of the primordium cells in the gall bladder. (**A**) Uniform manifold approximation and projection (UMAP) visualization of clustering results for gall bladder cells. (**B**) $R_{ACE2}$ in each cell cluster. (**C**) $R_{TMPRSS2}$ in each cell cluster. (**D**) ACE2 expression level in each cell cluster on the UMAP plot. (**E**) TMPRSS2 expression level in each cell cluster on the UMAP plot. (**F**) The expression distribution of ACE2 across each cell cluster. (**G**) The expression distribution of TMPRSS2 across each cell cluster.

The scRNA-seq data of the jejunum, ileum, duodenum, rectum, colon, esophagus, gall bladder, pancreatic islets, liver, and stomach from the digestive system were collected for the analysis. The primordium cells from the gall bladder contain 2.6% TMPRSS2-expressed cells and 2.2% ACE2-expressed cells (Figure 2, Table 2), which means the gall bladder is vulnerable to the SARS-CoV-2 infection. Moreover, the small intestine (jejunum, ileum, and duodenum), large intestine (rectum and colon), and the esophagus are identified as high-risk organs (Supplementary File: Figures S14–S19, Table 2). However, no cell clusters from the liver, stomach, and pancreatic islets data show high ACE2 and TMPRSS2 expression levels (Supplementary File: Figures S20–S22), which demonstrates a low infection risk.

### 3.3. Reproductive System

**Figure 3:**
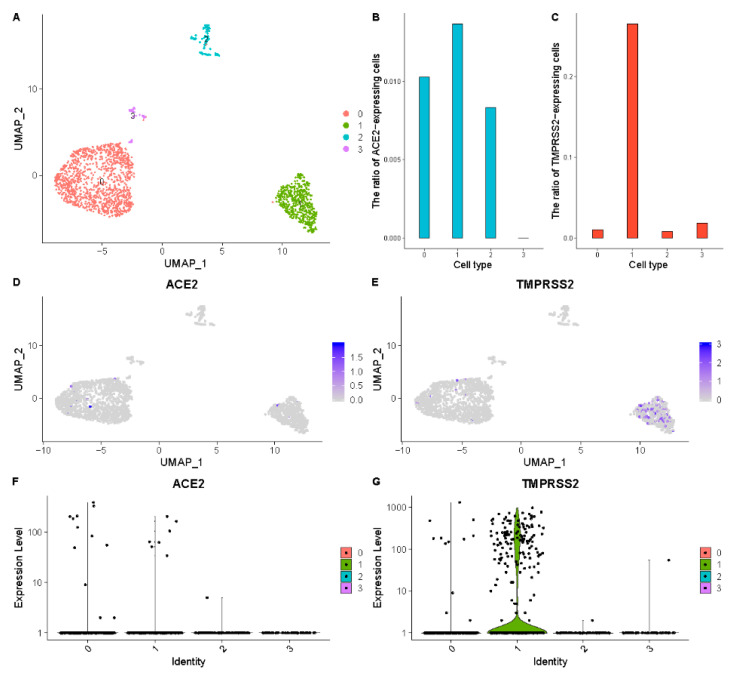
High ACE2 and TMPRSS2 expression levels of epithelial cells and ovarian somatic cells in the fallopian tube. (**A**) UMAP visualization of clustering results for the fallopian tube cells. (**B**) $R_{ACE2}$ in each cell cluster. (**C**) $R_{TMPRSS2}$ in each cell cluster. (**D**) ACE2 expression level in each cell cluster on the UMAP plot. (**E**) TMPRSS2 expression level in each cell cluster on the UMAP plot. (**F**) The expression distribution of ACE2 across each cell cluster. (**G**) The expression distribution of TMPRSS2 across each cell cluster.

The scRNA-seq data of the testis, fallopian tube, ovary, and uterus from the reproductive system were collected for analysis. The ratios of the TMPRSS2-expressed cells and the ACE2-expressed cells in the epithelial cells of the fallopian tube are 26.5% and 1.4%, respectively (Figure 3, Table 2), and the ovarian somatic cells contain 1% TMPRSS2-expressed cells and 1% ACE2-expressed cells, so the fallopian tube is identified as a high-risk organ. The testis is also identified as a high-risk organ because of the high expression level of TMPRSS2 and ACE2 (Supplementary File: Figure S23, Table 2). Low ratios of ACE2-expressed cells in the ovary and uterus mean the ovary and uterus are low infection risk organs (Supplementary File: Figures S24–S25).

### 3.4. Nervous System

The scRNA-seq of the substantia nigra and cortex, hippocampus, cerebellum, spinal cord, and neuronal epithelium from the nervous system were collected to infer the susceptibility of the organs. The analysis results show that ACE2 is expressed in the oligodendrocyte precursor cells and astrocytes of the substantia nigra and cortex with a high level, and TMPRSS2 is expressed as well. More specifically, astrocytes contain 1.9% ACE2-expressed cells, and oligodendrocyte precursor cells contain 1.6% ACE2-expressed cells (Supplementary File: Figure S26, Table 2). Therefore, the substantia nigra and cortex are predicted as high-risk tissues, and the brain is identified as a high-risk organ. For the analysis of other districts, cells from the hippocampus have low expression levels of TMPRSS2 and ACE2, and the cerebellum, spinal cord, and neuronal epithelium data show zero expression of TMPRSS2 and ACE2 (Supplementary File: Figures S27–S30), which demonstrates a low infection risk of these districts.

### 3.5. Circulatory System

The data of the heart, spleen, and artery from the circulatory system were collected to infer the susceptibility of these organs. The cardiomyocytes and cardiovascular progenitor cells from the heart contain 6.6% and 12.5% ACE2-expressed cells, respectively, and the TMPRSS2 is expressed in both cell clusters as well. Consequently, the heart is considered a high-risk organ (Supplementary File: Figure S31, Table 2). Nevertheless, almost no cells of the spleen, artery, and peripheral blood data show high TMPRSS2 and ACE2 expression levels, which means they are low-risk organs (Supplementary File: Figures S32–S34).

### 3.6. Urinary System

The scRNA-seq data of the kidney, ureter, and prostate from the urinary system were utilized for the data analysis. The analysis results of the kidney scRNA-seq data show high ACE2 and TMPRSS2 expression levels in the nephron epithelial cells, epithelial cells, endothelial cells, and mesangial cells. Particularly, the ratios of TMPRSS2-expressed are 10.7%, 9.6%, 12.8%, and 14.5%, respectively, and the ratios of ACE2-expressed are 2.7%, 2.7%, 2.7%, and 3.0%, respectively (Supplementary File: Figure S35, Table 2). Therefore, the kidney is considered a high-risk organ. In addition, the ACE2 is not expressed in the ureter and prostate cells (Supplementary File: Figures S36 and S37), and they are predicted to be low-risk infection organs.

### 3.7. Endocrine System, Motor System and Immune System

The susceptibility of organs from the endocrine system, immune system, and motor system was also considered. In the endocrine system, almost no cells from the thyroid gland data show high ACE2 and TMPRSS2 expression levels, and the thymus gland data contains no ACE2-expressed cells (Supplementary File: Figures S38 and S39). Hence, they are not high-risk organs. Likewise, few ACE2-expressed cells are found in the muscle from the motor system (Supplementary File: Figure S40). There is no ACE2 expression in the lymph nodes, tonsil (tonsil dendritic cells), and bone marrow data from the immune system (Supplementary File: Figures S41–S43), which means they are low-risk organs.

### 3.8. The Risk Levels of Susceptible Organs

**Figure 4:**
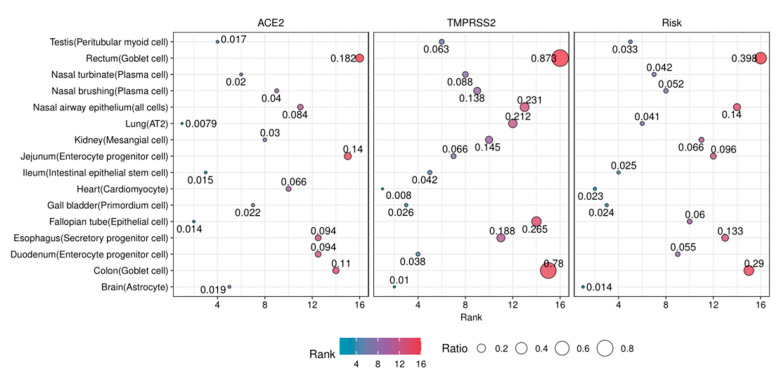
The risk levels of susceptible organs. The dot plot illustrates the rank of $R_{ACE2},R_{TMPRSS2}$ and R of 16 cell types. The sizes of the dot indicate the value of the ratio, and the colors indicate the rank of the ratio.

Based on TMPRSS2 and ACE2 expression levels, we grouped the susceptible organs into three risk levels. According to the clinical implication, the lung should be the highest risk organ to SARS-CoV-2 infection. Interestingly, the large intestine (colon and rectum), esophagus, and nose (nasal airway epithelium) are the most susceptible organs, and the result indicates SARS-CoV-2 mainly attacks the respiratory system and the digestive system (Table 3, Figure 4). The kidney, small intestine (duodenum and jejunum), and fallopian tube are susceptible organs with moderate risk (Table 3, Figure 4). In addition, the testis, gall bladder, brain (substantia nigra and cortex), and heart are identified to be potentially susceptible organs (Table 3, Figure 4).

## 4. Discussion

ACE2 has been reported to show a significant overexpression in COVID-19 patients and is positively correlated with the expression of some other SARS-COV-2 host invasion genes [27]. Compared with current research on organ susceptibility to COVID-19 using scRNA-seq data, the advantages of our research are mainly reflected in the following aspects. Firstly, we presented a more comprehensive and rigorous analysis of 31 human organs from nine systems based on ACE2 and TMPRSS2 expression. Then, we obtained breakthrough results that the gall bladder and fallopian tube are vulnerable to SARS-CoV-2 infection. Finally, we classified susceptible organs to different risk levels and found that the respiratory system, digestive system, and urinary system are at the top-risk level for SARS-CoV-2 infection.

At present, nucleic acid testing is the most extensive testing technology for mild patients and suspected cases. The screening samples for nucleic acid detection are mostly from deep cough sputum, oropharyngeal swabs, or nasopharyngeal swabs. However, this detection technology may cause false-negative results. In some COVID-19 cases, patients have gastrointestinal (GI) symptoms, such as anorexia, vomiting and diarrhea, without any respiratory symptoms in the initial stages of the disease [70,71]. Our results verify that the esophagus and intestines (rectum, ileum, jejunum, and duodenum) are also the main sites of SARS-CoV-2 infection in the human body. Previous studies have reported that SARS-CoV-2 can be detected in the feces of infected persons, suggesting that there may be food transmission and fecal–oral transmission during the spread of SARS-CoV-2 [72,73]. Therefore, epidemic prevention experts recommend paying attention to good personal hygiene and food handling practices. In addition, neurological symptoms such as headache, loss of sense of smell, amblyopia, weakness, and myalgia were described in some COVID-19 patients [5,74,75]. Our results show that the expression ratio of ACE2 in the cortex and substantia nigra of healthy people is higher than that in AT2 cells, which indicates that SARS-CoV-2 can also invade the nervous system. Therefore, the symptoms of some organs of the digestive system and nervous system can be used as the basis for preliminary judgment of the patient’s condition.

Previous studies have reported PCR fragments of the coronavirus detected in the blood and urine of COVID-19 patients, as well as nephritis histological analysis of the autopsy results, indicating that SARS-CoV-2 causes acute tubular disease [76,77]. The renal tropism of SARS-CoV-2 revealed by the high expression of ACE2 and TMPRSS2 in the kidney may explain the renal injury in COVID-19 patients. The results show that the heart is also a susceptible organ, indicating that SARS-CoV-2 may affect the normal operation of the cardiovascular system. In fact, SARS-CoV-2 infection has been associated with cardiovascular complications, such as acute myocardial injury, and myocarditis, which increases mortality [76,78]. Besides, patients with cardiovascular and cerebrovascular diseases are prone to COVID-19-related complications due to their relatively fragile cardiovascular system [76]. Therefore, it is necessary to pay attention to the pathological changes of the urinary system and circulatory system organs when treating COVID-19 patients. Moreover, it should be noted that, according to the results of this study, SARS-CoV-2 may affect the reproductive organs. We suggest that patients should contraception during antiviral treatment, and it is recommended to continue contraception after a period of treatment.

However, the cell entry mechanisms of SARS-CoV-2 are not fully understood, and the invasion process may also be highly related to other genes than ACE2 and TMPRSS2. Therefore, our results need to be further confirmed by clinical observations and biological experiments. Due to the limitation of data collection, the susceptibility of some human organs remains to be analyzed. Moreover, whether some chronic diseases or cancer patients are more susceptible to SARS-CoV-2 infection is also worth exploring.

## 5. Conclusions

The clinical symptoms of patients infected by SARS-CoV-2 are mainly manifested in the respiratory system and digestive system, including cough, shortness of breath, dyspnea, and diarrhea. However, some patients also developed symptoms such as heart damage and kidney failure, indicating that the virus affected the normal function of the circulatory and urinary systems. We investigated the susceptibility of the organs and tissues in various human systems based on the scRNA-seq data analysis. In detail, 31 organs from nine major human systems were considered, out of which 11 organs were identified to be susceptible to the virus. Moreover, we classified these susceptible organs into three levels in terms of their risk, which provide novel ideas for the follow-up detection of the virus, treatment, and the monitoring of recrudescence.

Through the assessment of the susceptibility of human organs to SARS-CoV-2 infection and the accurate judgment of the viral invasion to organs, we can further understand the pathophysiology of the disease and help determine the prognostic utility of clinical and laboratory parameters related to COVID-19, which will lead to the development of more precise and effective management methods for the disease.

## Figures and Tables

**Table 1:** Human organs available for single-cell RNA sequencing (scRNA-seq) data analysis.

Human Systems	Human Organs
**Digestive system**	Esophagus [57]
	Small intestine (jejunum, ileum, andduodenum) [58]
	Large intestine (rectum, colon)
	Stomach [58]
	Liver [58]
	Gall bladder [58]
	Pancreatic islets [59]
**Nervous system**	Brain (substantia nigra and cortex, neuronal epithelium, and hippocampus) [60,61]
	Cerebellum [58]
	Spinal cord [58]
**Reproductive system**	Ovary [62]
	Fallopian tube [63]
	Uterus [58]
	Testis [64]
**Motor system**	Muscle [58]
**Respiratory system**	Nose (nasal brushing epithelial cells, nasal turbinate epithelial cells, and nasal airway epithelium) [65]
	Bronchus [65]
	Lung [66]
	Trachea [58]
**Circulatory system**	Peripheral blood [58]
	Heart [67]
	Artery [58]
	Spleen [57]
**Urinary system**	Kidney [58]
	Ureter [58]
	Prostate [58]
**Immune system**	Tonsil (tonsil dendritic cells) [68]
	Bone marrow [58]
	Lymph nodes [69]
**Endocrine system**	Thyroid [58]
	Thymus [58]

**Table 2:** Cell types with high expression levels of ACE2 and TMPRSS2.

Systems	Organs	Cell Types	$R_{ACE2}$	$R_{TMPRSS2}$
**Respiratory system**	Lung	AT2 cells	0.79%	21.20%
	Nose (nasal turbinate epithelial cells)	Mesenchymal stromal cells	1.70%	8.50%
		Plasma cells	2.00%	8.80%
	Nose (nasal brushing epithelial cells)	Plasma cells	4.00%	13.80%
	Nose (nasal airway epithelial cells)	Nasal airway epithelial cells	8.40%	23.10%
**Digestive system**	Gall bladder	Primordium cell	2.20%	2.60%
	Small intestine (jejunum)	Enterocyte progenitor cell	14.00%	6.60%
		Goblet cell	9.10%	2.60%
	Small intestine (ileum)	Intestinal epithelial stem cell	1.50%	4.20%
		Enterocyte progenitor cell	2.40%	3.60%
	Small intestine (duodenum)	LGR5+ stem cell	5.20%	4.50%
		Intestinal epithelial stem cell	3.90%	5.80%
		Enterocyte progenitor cell	4.40%	6.80%
		Tuft progenitor cell	7.40%	6.60%
		Enteroendocrine cell	9.40%	3.80%
	Large intestine (rectum)	Goblet progenitor cell	2.80%	62.50%
		MKI67+ progenitor cell	10.30%	75.40%
		Enterocyte	13.20%	77.50%
		Goblet cell	18.20%	87.30%
	Large intestine (colon)	Enterocyte	5.70%	52.10%
		Goblet cell	11.00%	78.00%
	Esophagus	Secretory progenitor cell	9.40%	18.80%
**Nervous system**	Brain (substantia nigra and cortex)	Oligodendrocyte precursor cell	1.60%	0.90%
		Astrocyte	1.90%	1.00%
**Reproductive system**	Fallopian tube	Epithelial cells	1.40%	26.50%
		Ovarian somatic cell	1.00%	1.00%
	Testis	Spermatogonium	1.70%	4.50%
		Peritubular myoid cell	1.70%	6.30%
		Testis somatic cell	2.10%	4.30%
		Spermatogonial stem cell	1.40%	2.10%
**Circulatory system**	Heart	Cardiomyocyte	6.60%	0.80%
		Cardiovascular progenitor cell	12.50%	0.40%
**Urinary system**	Kidney	Nephron epithelial cell	2.70%	10.70%
		Epithelial cell	2.70%	9.60%
		Endothelial cell	2.70%	12.80%
		Mesangial cell	3.00%	14.50%

**Table 3:** Risk level of organs to SARS-CoV-2 infection.

Organs	Systems	Cell Types	*R*	Risk Levels
Large intestine (rectum)	Digestive system	Goblet cell	0.398	1
Large intestine (colon)	Digestive system	Goblet cell	0.290	1
Nose (nasal airway epithelium)	Respiratory system	All cells	0.140	1
Esophagus	Digestive system	Secretory progenitor cell	0.133	1
Small intestine (jejunum)	Digestive system	Enterocyte progenitor cell	0.096	2
Kidney	Urinary system	Mesangial cell	0.066	2
Fallopian tube	Reproductive system	Epithelial cell	0.060	2
Small intestine (duodenum)	Digestive system	Enterocyte progenitor cell	0.055	2
Nose (nasal brushing epithelial cells)	Respiratory system	Plasma cell	0.052	2
Nose (nasal turbinate epithelial cells)	Respiratory system	Plasma cell	0.042	3
Lung	Respiratory system	AT2	0.041	3
Testis	Reproductive system	Peritubular myoid cell	0.033	3
Small intestine (ileum)	Digestive system	Intestinal epithelial stem cell	0.025	3
Gall bladder	Digestive system	Primordium cell	0.024	3
Heart	Circulatory system	Cardiomyocyte	0.023	3
Brain (substantia nigra and cortex)	Nervous system	Astrocyte	0.014	3

## Data Availability

The data were downloaded from the Tissue Stability Cell Atlas, GEO database, and the details of the data resource can be seen in Supplementary File Table S1.

## Supplementary Materials

The following are available online at https://www.mdpi.com/1660-4601/18/1/284/s1, Figure S1: The lung scRNA-seq data analysis results (donor 1). (A) UMAP visualization of clustering results for the lung cells. (B) The ratio of ACE2-expressed cells in each cell cluster. (C) The ratio of TMPRSS2-expressed cells in each cell cluster. (D) ACE2 expression level in each cell cluster on the UMAP plot. (E) TMPRSS2 expression level in each cell cluster on the UMAP plot. (F) The expression distribution of ACE2 across each cell cluster. (G) The expression distribution of TMPRSS2 across each cell cluster, Figure S2: The lung scRNA-seq data analysis results (donor 2). (A) UMAP visualization of clustering results for the lung cells. (B) The ratio of ACE2-expressed cells in each cell cluster. (C) The ratio of TMPRSS2-expressed cells in each cell cluster. (D) ACE2 expression level in each cell cluster on the UMAP plot. (E) TMPRSS2 expression level in each cell cluster on the UMAP plot. (F) The expression distribution of ACE2 across each cell cluster. (G) The expression distribution of TMPRSS2 across each cell cluster, Figure S3: The lung scRNA-seq data analysis results (donor 3). (A) UMAP visualization of clustering results for the lung cells. (B) The ratio of ACE2-expressed cells in each cell cluster. (C) The ratio of TMPRSS2-expressed cells in each cell cluster. (D) ACE2 expression level in each cell cluster on the UMAP plot. (E) TMPRSS2 expression level in each cell cluster on the UMAP plot. (F) The expression distribution of ACE2 across each cell cluster. (G) The expression distribution of TMPRSS2 across each cell cluster, Figure S4: The lung scRNA-seq data analysis results (donor 4). (A) UMAP visualization of clustering results for the lung cells. (B) The ratio of ACE2-expressed cells in each cell cluster. (C) The ratio of TMPRSS2-expressed cells in each cell cluster. (D) ACE2 expression level in each cell cluster on the UMAP plot. (E) TMPRSS2 expression level in each cell cluster on the UMAP plot. (F) The expression distribution of ACE2 across each cell cluster. (G) The expression distribution of TMPRSS2 across each cell cluster, Figure S5: The lung scRNA-seq data analysis results (donor 5). (A) UMAP visualization of clustering results for the lung cells. (B) The ratio of ACE2-expressed cells in each cell cluster. (C) The ratio of TMPRSS2-expressed cells in each cell cluster. (D) ACE2 expression level in each cell cluster on the UMAP plot. (E) TMPRSS2 expression level in each cell cluster on the UMAP plot. (F) The expression distribution of ACE2 across each cell cluster. (G) The expression distribution of TMPRSS2 across each cell cluster, Figure S6: The lung scRNA-seq data analysis results (donor 6). (A) UMAP visualization of clustering results for the lung cells. (B) The ratio of ACE2-expressed cells in each cell cluster. (C) The ratio of TMPRSS2-expressed cells in each cell cluster. (D) ACE2 expression level in each cell cluster on the UMAP plot. (E) TMPRSS2 expression level in each cell cluster on the UMAP plot. (F) The expression distribution of ACE2 across each cell cluster. (G) The expression distribution of TMPRSS2 across each cell cluster, Figure S7: The lung scRNA-seq data analysis results (donor 7). (A) UMAP visualization of clustering results for the lung cells. (B) The ratio of ACE2-expressed cells in each cell cluster. (C) The ratio of TMPRSS2-expressed cells in each cell cluster. (D) ACE2 expression level in each cell cluster on the UMAP plot. (E) TMPRSS2 expression level in each cell cluster on the UMAP plot. (F) The expression distribution of ACE2 across each cell cluster. (G) The expression distribution of TMPRSS2 across each cell cluster, Figure S8: The lung scRNA-seq data analysis results (donor 8). (A) UMAP visualization of clustering results for the lung cells. (B) The ratio of ACE2-expressed cells in each cell cluster. (C) The ratio of TMPRSS2-expressed cells in each cell cluster. (D) ACE2 expression level in each cell cluster on the UMAP plot. (E) TMPRSS2 expression level in each cell cluster on the UMAP plot. (F) The expression distribution of ACE2 across each cell cluster. (G) The expression distribution of TMPRSS2 across each cell cluster, Figure S9: High ACE2 and TMPRSS2 expression levels of the mesenchymal stromal cells, plasma cells in the nasal turbinate epithelial cells. (A) UMAP visualization of clustering results for the nasal turbinate epithelial cells. (B) The ratio of ACE2-expressed cells in each cell cluster. (C) The ratio of TMPRSS2-expressed cells in each cell cluster. (D) ACE2 expression level in each cell cluster on the UMAP plot. (E) TMPRSS2 expression level in each cell cluster on the UMAP plot. (F) The expression distribution of ACE2 across each cell cluster. (G) The expression distribution of TMPRSS2 across each cell cluster, Figure S10: High ACE2 and TMPRSS2 expression levels of the mesenchymal stromal cells, plasma cells in the nasal brushing epithelial cells. (A) UMAP visualization of clustering results for the nasal brushing epithelial cells. (B) The ratio of ACE2-expressed cells in each cell cluster. (C) The ratio of TMPRSS2-expressed cells in each cell cluster. (D) ACE2 expression level in each cell cluster on the UMAP plot. (E) TMPRSS2 expression level in each cell cluster on the UMAP plot. (F) The expression distribution of ACE2 across each cell cluster. (G) The expression distribution of TMPRSS2 across each cell cluster, Figure S11: High ACE2 and TMPRSS2 expression levels in the nasal airway epithelial cells. (A) UMAP visualization of clustering results for the airway epithelial cells. (B) The ratio of ACE2-expressed cells in each cell cluster. (C) The ratio of TMPRSS2-expressed cells in each cell cluster. (D) ACE2 expression level in each cell cluster on the UMAP plot. (E) TMPRSS2 expression level in each cell cluster on the UMAP plot. (F) The expression distribution of ACE2 across each cell cluster. (G) The expression distribution of TMPRSS2 across each cell cluster, Figure S12: The bronchus scRNA-seq data analysis results. (A) UMAP visualization of clustering results for the bronchus cells. (B) The ratio of ACE2-expressed cells in each cell cluster. (C) The ratio of TMPRSS2-expressed cells in each cell cluster. (D) ACE2 expression level in each cell cluster on the UMAP plot. (E) TMPRSS2 expression level in each cell cluster on the UMAP plot. (F) The expression distribution of ACE2 across each cell cluster. (G) The expression distribution of TMPRSS2 across each cell cluster, Figure S13: The trachea scRNA-seq data analysis results. (A) UMAP visualization of clustering results for the trachea cells. (B) The ratio of ACE2-expressed cells in each cell cluster. (C) The ratio of TMPRSS2-expressed cells in each cell cluster. (D) ACE2 expression level in each cell cluster on the UMAP plot. (E) TMPRSS2 expression level in each cell cluster on the UMAP plot. (F) The expression distribution of ACE2 across each cell cluster. (G) The expression distribution of TMPRSS2 across each cell cluster, Figure S14: High ACE2 and TMPRSS2 expression level of enterocyte progenitor cells and goblet cells in the jejunum. (A)UMAP visualization of clustering results for jejunum cells. (B)The ratio of ACE2-expressed cells in each cell cluster. (C)The ratio of TMPRSS2-expressed cells in each cell cluster. (D)ACE2 expression level in each cell cluster on the UMAP plot. (E)TMPRSS2 expression level in each cell cluster on the UMAP plot. (F)The expression distribution of ACE2 across each cell cluster. (G)The expression distribution of TMPRSS2 across each cell cluster, Figure S15: High ACE2 and TMPRSS2 expression level of the intestinal epithelial stem cells and enterocyte progenitor cells in the ileum. (A) UMAP visualization of clustering results for ileum cells. (B) The ratio of ACE2-expressed cells in each cell cluster. (C) The ratio of TMPRSS2-expressed cells in each cell cluster. (D) ACE2 expression level in each cell cluster on the UMAP plot. (E) TMPRSS2 expression level in each cell cluster on the UMAP plot. (F) The expression distribution of ACE2 across each cell cluster. (G) The expression distribution of TMPRSS2 across each cell cluster, Figure S16: High ACE2 and TMPRSS2 expression level of the intestinal LGR5+ stem cells, epithelial stem cells, enterocyte progenitor cells, tuft progenitor cells, and enteroendocrine cells in the duodenum. (A) UMAP visualization of clustering results for duodenum cells. (B) The ratio of ACE2-expressed cells in each cell cluster. (C) The ratio of TMPRSS2-expressed cells in each cell cluster. (D) ACE2 expression level in each cell cluster on the UMAP plot. (E) TMPRSS2 expression level in each cell cluster on the UMAP plot. (F) The expression distribution of ACE2 across each cell cluster. (G) The expression distribution of TMPRSS2 across each cell cluster, Figure S17: High ACE2 and TMPRSS2 expression level of goblet progenitor cells, MKI67+ progenitor cells, enterocytes, and goblet cells in the rectum. (A) UMAP visualization of clustering results for rectum cells. (B) The ratio of ACE2-expressed cells in each cell cluster. (C) The ratio of TMPRSS2-expressed cells in each cell cluster. (D) ACE2 expression level in each cell cluster on the UMAP plot. (E) TMPRSS2 expression level in each cell cluster on the UMAP plot. (F) The expression distribution of ACE2 across each cell cluster. (G) The expression distribution of TMPRSS2 across each cell cluster, Figure S18: High ACE2 and TMPRSS2 expression level of the enterocytes and goblet cells in the colon. (A) UMAP visualization of clustering results for colon cells. (B) The ratio of ACE2-expressed cells in each cell cluster. (C) The ratio of TMPRSS2-expressed cells in each cell cluster. (D) ACE2 expression level in each cell cluster on the UMAP plot. (E) TMPRSS2 expression level in each cell cluster on the UMAP plot. (F) The expression distribution of ACE2 across each cell cluster. (G) The expression distribution of TMPRSS2 across each cell cluster, Figure S19: High ACE2 and TMPRSS2 expression level of the secretory progenitor cells in the esophagus. (A) UMAP visualization of clustering results for esophagus cells. (B) The ratio of ACE2-expressed cells in each cell cluster. (C) The ratio of TMPRSS2-expressed cells in each cell cluster. (D) ACE2 expression level in each cell cluster on the UMAP plot. (E) TMPRSS2 expression level in each cell cluster on the UMAP plot. (F) The expression distribution of ACE2 across each cell cluster. (G) The expression distribution of TMPRSS2 across each cell cluster, Figure S20: The liver scRNA-seq data analysis results. (A) UMAP visualization of clustering results for liver cells. (B) The ratio of ACE2-expressed cells in each cell cluster. (C) The ratio of TMPRSS2-expressed cells in each cell cluster. (D) ACE2 expression level in each cell cluster on the UMAP plot. (E) TMPRSS2 expression level in each cell cluster on the UMAP plot. (F) The expression distribution of ACE2 across each cell cluster. (G) The expression distribution of TMPRSS2 across each cell cluster, Figure S21: The stomach scRNA-seq data analysis results. (A) UMAP visualization of clustering results for stomach cells. (B) The ratio of ACE2-expressed cells in each cell cluster. (C) The ratio of TMPRSS2-expressed cells in each cell cluster. (D) ACE2 expression level in each cell cluster on the UMAP plot. (E) TMPRSS2 expression level in each cell cluster on the UMAP plot. (F) The expression distribution of ACE2 across each cell cluster. (G) The expression distribution of TMPRSS2 across each cell cluster, Figure S22: The pancreatic islets scRNA-seq data analysis results. (A) UMAP visualization of clustering results for pancreatic islets cells. (B) The ratio of ACE2-expressed cells in each cell cluster. (C) The ratio of TMPRSS2-expressed cells in each cell cluster. (D) ACE2 expression level in each cell cluster on the UMAP plot. (E) TMPRSS2 expression level in each cell cluster on the UMAP plot. (F) The expression distribution of ACE2 across each cell cluster. (G) The expression distribution of TMPRSS2 across each cell cluster, Figure S23: High ACE2 and TMPRSS2 expression level of spermatogonium, peritubular myoid cells, testis somatic cells, and spermatogonial stem cells in the testis. (A) UMAP visualization of clustering results for testis cells. (B) The ratio of ACE2-expressed cells in each cell cluster. (C) The ratio of TMPRSS2-expressed cells in each cell cluster. (D) ACE2 expression level in each cell cluster on the UMAP plot. (E) TMPRSS2 expression level in each cell cluster on the UMAP plot. (F) The expression distribution of ACE2 across each cell cluster. (G) The expression distribution of TMPRSS2 across each cell cluster, Figure S24: The ovary scRNA-seq data analysis results. (A) UMAP visualization of clustering results for ovary cells. (B) The ratio of ACE2-expressed cells in each cell cluster. (C) ACE2 expression level in each cell cluster on the UMAP plot. (D) The expression distribution of ACE2 across each cell cluster, Figure S25: The uterus scRNA-seq data analysis results. (A) UMAP visualization of clustering results for uterus cells. (B) The ratio of ACE2-expressed cells in each cell cluster. (C) ACE2 expression level in each cell cluster on the UMAP plot. (D) The expression distribution of ACE2 across each cell cluster, Figure S26: High ACE2 and TMPRSS2 expression level of oligodendrocyte precursor cells and astrocytes in the substantia nigra and cortex. (A) UMAP visualization of clustering results for the substantia nigra and cortex cells. (B) The ratio of ACE2-expressed cells in each cell cluster. (C) The ratio of TMPRSS2-expressed cells in each cell cluster. (D) ACE2 expression level in each cell cluster on the UMAP plot. (E) TMPRSS2 expression level in each cell cluster on the UMAP plot. (F) The expression distribution of ACE2 across each cell cluster. (G) The expression distribution of TMPRSS2 across each cell cluster, Figure S27: The hippocampus scRNA-seq data analysis results. (A) UMAP visualization of clustering results for hippocampus cells. (B) The ratio of ACE2-expressed cells in each cell cluster. (C) The ratio of TMPRSS2-expressed cells in each cell cluster. (D) ACE2 expression level in each cell cluster on the UMAP plot. (E) TMPRSS2 expression level in each cell cluster on the UMAP plot. (F) The expression distribution of ACE2 across each cell cluster. (G) The expression distribution of TMPRSS2 across each cell cluster, Figure S28: UMAP visualization of clustering results for the cerebellum cells, Figure S29: UMAP visualization of clustering results for the spinal cord cells, Figure S30: UMAP visualization of clustering results for the neuronal epithelium cells, Figure S31: High ACE2 and TMPRSS2 expression level of the cardiomyocytes and cardiovascular progenitor cells in the heart. (A) UMAP visualization of clustering results for the heart cells. (B) The ratio of ACE2-expressed cells in each cell cluster. (C) The ratio of TMPRSS2-expressed cells in each cell cluster. (D) ACE2 expression level in each cell cluster on the UMAP plot. (E) TMPRSS2 expression level in each cell cluster on the UMAP plot. (F) The expression distribution of ACE2 across each cell cluster. (G) The expression distribution of TMPRSS2 across each cell cluster, Figure S32: The spleen scRNA-seq data analysis results. (A) UMAP visualization of clustering results for the spleen cells. (B) The ratio of ACE2-expressed cells in each cell cluster. (C) The ratio of TMPRSS2-expressed cells in each cell cluster. (D) ACE2 expression level in each cell cluster on the UMAP plot. (E) TMPRSS2 expression level in each cell cluster on the UMAP plot. (F) The expression distribution of ACE2 across each cell cluster. (G) The expression distribution of TMPRSS2 across each cell cluster, Figure S33: The artery scRNA-seq data analysis results. (A) UMAP visualization of clustering results for the spleen cells. (B) The ratio of ACE2-expressed cells in each cell cluster. (C) ACE2 expression level in each cell cluster on the UMAP plot. (D) The expression distribution of ACE2 across each cell cluster, Figure S34: UMAP visualization of clustering results for the peripheral blood cells, Figure S35: High ACE2 and TMPRSS2 expression level of the nephron epithelial cells, epithelial cells, endothelial cells, and mesangial cells in the kidney. (A) UMAP visualization of clustering results for the kidney cells. (B) The ratio of ACE2-expressed cells in each cell cluster. (C) The ratio of TMPRSS2-expressed cells in each cell cluster. (D) ACE2 expression level in each cell cluster on the UMAP plot. (E) TMPRSS2 expression level in each cell cluster on the UMAP plot. (F) The expression distribution of ACE2 across each cell cluster. (G) The expression distribution of TMPRSS2 across each cell cluster, Figure S36: UMAP visualization of clustering results for the ureter cells, Figure S37: UMAP visualization of clustering results for the prostate cells, Figure S38: The thyroid gland scRNA-seq data analysis results. (A) UMAP visualization of clustering results for the thyroid gland cells. (B) The ratio of ACE2-expressed cells in each cell cluster. (C) The ratio of TMPRSS2-expressed cells in each cell cluster. (D) ACE2 expression level in each cell cluster on the UMAP plot. (E) TMPRSS2 expression level in each cell cluster on the UMAP plot. (F) The expression distribution of ACE2 across each cell cluster. (G) The expression distribution of TMPRSS2 across each cell cluster, Figure S39: UMAP visualization of clustering results for the thymus gland cells, Figure S40: The muscle scRNA-seq data analysis results. (A) UMAP visualization of clustering results for the muscle cells. (B) The ratio of ACE2-expressed cells in each cell cluster. (C) ACE2 expression level in each cell cluster on the UMAP plot. (D) The expression distribution of ACE2 across each cell cluster, Figure S41: UMAP visualization of clustering results for the lymph nodes cells, Figure S42: UMAP visualization of clustering results for the tonsil dendritic cells, Figure S43: The bone marrow scRNA-seq data analysis results. (A) UMAP visualization of clustering results for the bone marrow cells. (B) The ratio of ACE2-expressed cells in each cell cluster. (C) ACE2 expression level in each cell cluster on the UMAP plot. (D) The expression distribution of ACE2 across each cell cluster, Table S1: The data sources of organs and tissues.
