# Supplementary material for: The scRNA-seq Expression Profiling of the Receptor ACE2 and the Cellular Protease TMPRSS2 Reveals Human Organs Susceptible to SARS-CoV-2 Infection
Source: Int J Environ Res Public Health. 2021 Jan 2;18(1):284. doi: 10.3390/ijerph18010284 (PMC7794913; doi:10.3390/ijerph18010284)
Supplement: Supplementary file 1 [file ijerph-18-00284-s001.pdf]

# Supplementary materials

Table S1. The data sources of organs and tissues.

| Human systems | Human tissues | Data sources |
|---|---|---|
| **Digestive system** | Esophagus | https://www.tissuestabilitycellatlas.org/ |
| | Small intestine (Jejunum) | GEO: GSE134355 |
| | Small intestine (Ileum) | GEO: GSE134355 |
| | Small intestine (Duodenum) | GEO: GSE134355 |
| | Large intestine (Rectum) | GEO: GSE125970 |
| | Large intestine (Colon) | GEO: GSE125970 |
| | Stomach | GEO: GSE134355 |
| | Liver | GEO: GSE134355 |
| | Gall bladder | GEO: GSE134355 |
| | Pancreatic islets | GEO: GSE114297 |
| **Nervous system** | Brain (Substantia nigra and cortex) | GEO: GSE140231 |
| | Brain (Neuronal epithelium) | GEO: GSE81475 |
| | Brain (Hippocampus) | GEO: GSE119212 |
| | Cerebellum | GEO: GSE134355 |
| | Spinal cord | GEO: GSE134355 |
| **Reproductive system** | Ovary | GEO: GSE118127 |
| | Fallopian tube | GEO: GSE139079 |
| | Uterus | GEO: GSE134355 |
| | Testis | GEO: GSE112013 |
| **Motor system** | Muscle | GEO: GSE134355 |
| **Respiratory system** | Nose (Nasal brushing epithelial cells) | GEO: GSE121600 |
| | Nose (Nasal turbinate epithelial cells) | GEO: GSE121600 |
| | Nose (nasal airway epithelium) | GEO: GSE103518 |
| | Bronchus | GEO: GSE121600 |
| | Lung | GEO: GSE122960 |
| | Trachea | GEO: GSE134355 |
| **Circulatory system** | Peripheral blood | GEO: GSE134355 |
| | Heart | GEO: GSE106118 |
| | Artery | GEO: GSE134355 |
| | Spleen | https://www.tissuestabilitycellatlas.org/ |
| **Urinary system** | Kidney | GEO: GSE134355 |
| | Ureter | GEO: GSE134355 |
| | Prostate | GEO: GSE134355 |
| **Immune system** | Tonsil (Tonsil dendritic cells) | GEO: GSE115006 |
| | Bone marrow | GEO: GSE134355 |
| | Lymph nodes | GEO: GSE124494 |
| **Endocrine system** | Thyroid | GEO: GSE134355 |
| | Thymus | GEO: GSE134355 |

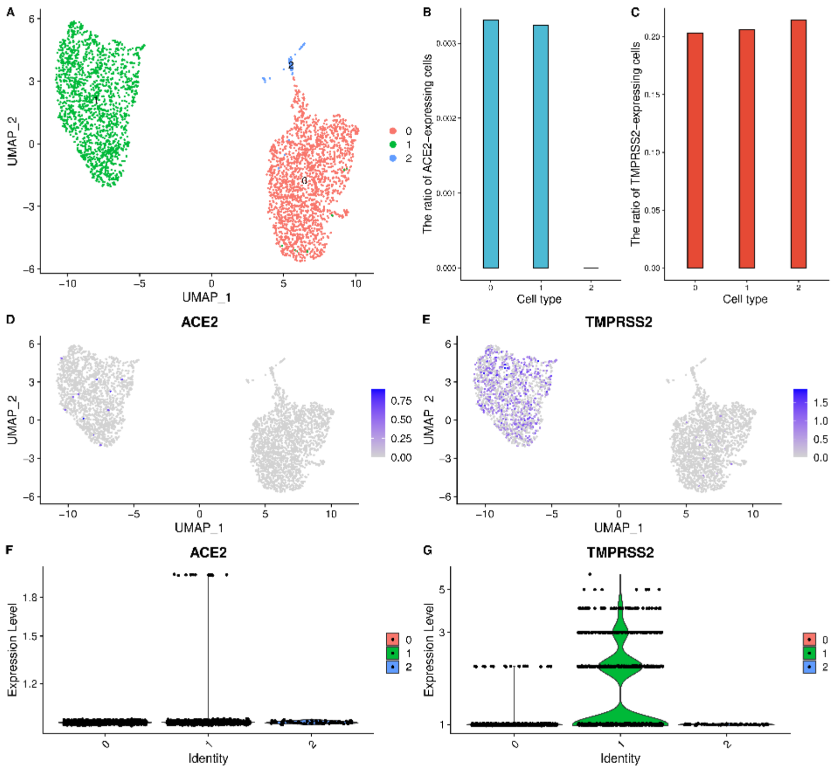


Fig. S1. The lung scRNA-seq data analysis results (donor 1). (A) UMAP visualization of clustering results for the lung cells. (B) The ratio of ACE2-expressed cells in each cell cluster. (C) The ratio of TMPRSS2-expressed cells in each cell cluster. (D) ACE2 expression level in each cell cluster on the UMPA plot. (E) TMPRSS2 expression level in each cell cluster on the UMPA plot. (F) The expression distribution of ACE2 across each cell cluster. (G) The expression distribution of TMPRSS2 across each cell cluster.

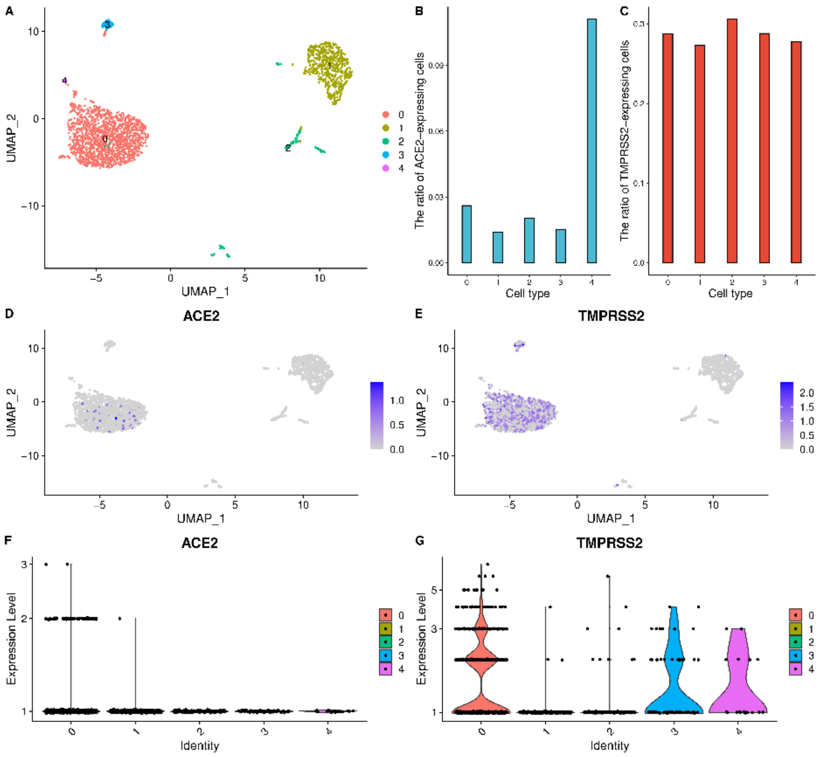


Fig. S2. The lung scRNA-seq data analysis results (donor 2). (A) UMAP visualization of clustering results for the lung cells. (B) The ratio of ACE2-expressed cells in each cell cluster. (C) The ratio of TMPRSS2-expressed cells in each cell cluster. (D) ACE2 expression level in each cell cluster on the UMPA plot. (E) TMPRSS2 expression level in each cell cluster on the UMPA plot. (F) The expression distribution of ACE2 across each cell cluster. (G) The expression distribution of TMPRSS2 across each cell cluster.

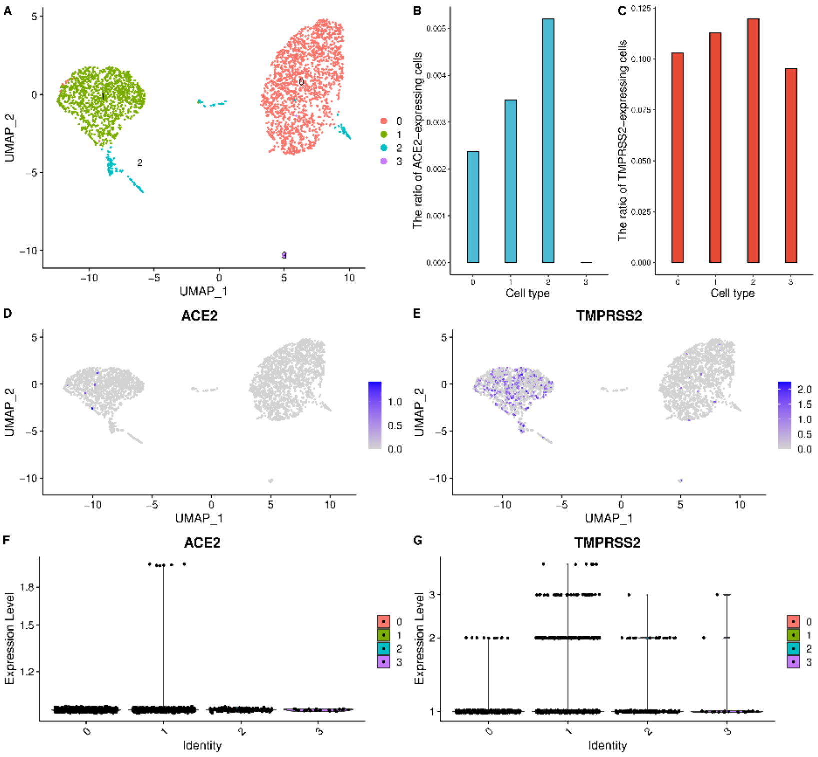


Fig. S3. The lung scRNA-seq data analysis results (donor 3). (A) UMAP visualization of clustering results for the lung cells. (B) The ratio of ACE2-expressed cells in each cell cluster. (C) The ratio of TMPRSS2-expressed cells in each cell cluster. (D) ACE2 expression level in each cell cluster on the UMPA plot. (E) TMPRSS2 expression level in each cell cluster on the UMPA plot. (F) The expression distribution of ACE2 across each cell cluster. (G) The expression distribution of TMPRSS2 across each cell cluster.

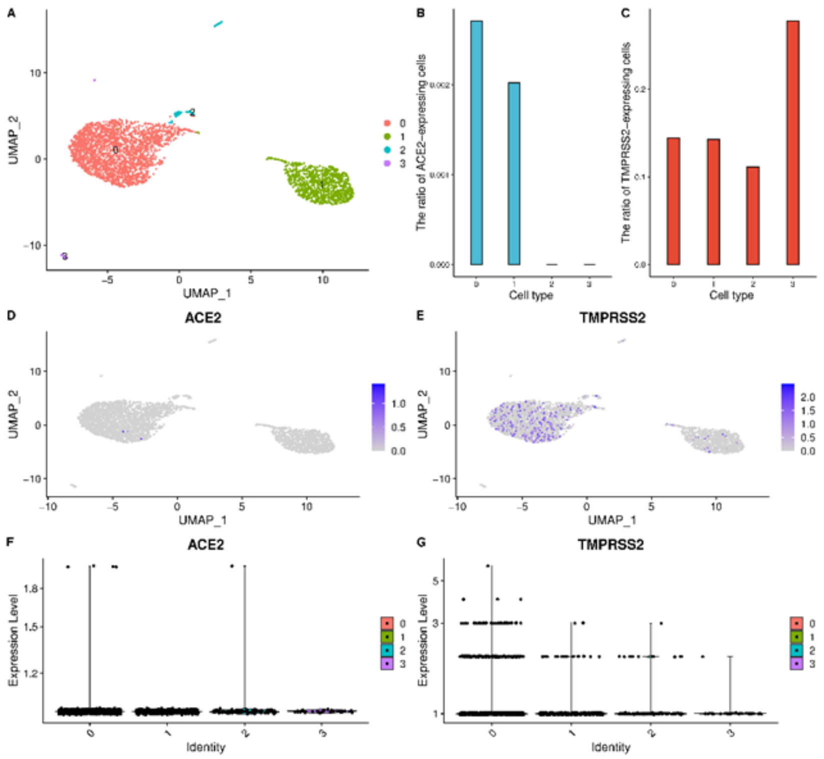


Fig. S4. The lung scRNA-seq data analysis results (donor 4). (A) UMAP visualization of clustering results for the lung cells. (B) The ratio of ACE2-expressed cells in each cell cluster. (C) The ratio of TMPRSS2-expressed cells in each cell cluster. (D) ACE2 expression level in each cell cluster on the UMPA plot. (E) TMPRSS2 expression level in each cell cluster on the UMPA plot. (F) The expression distribution of ACE2 across each cell cluster. (G) The expression distribution of TMPRSS2 across each cell cluster.

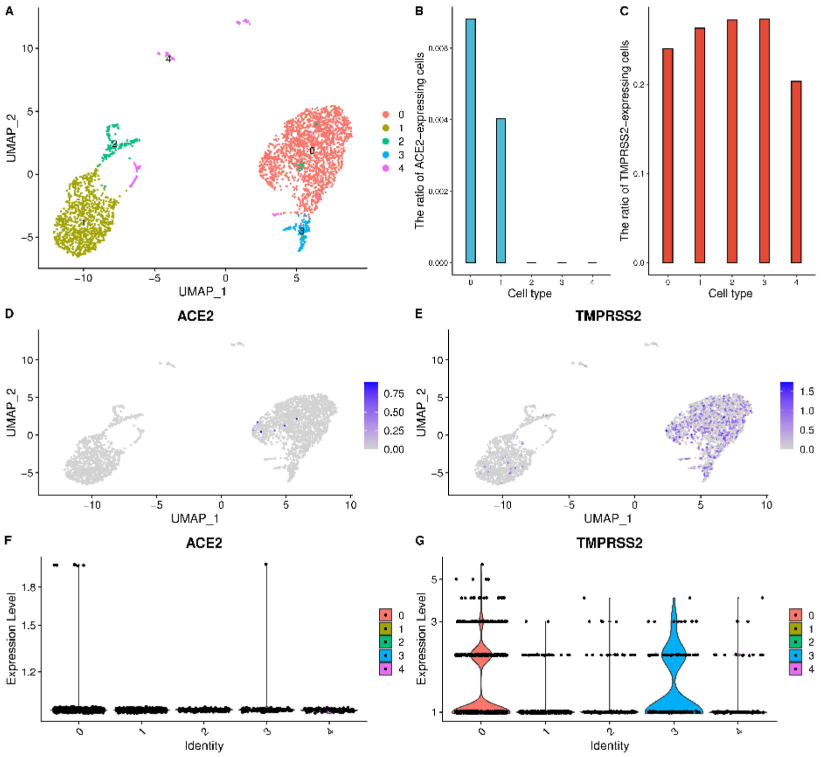

Fig. S5. The lung scRNA-seq data analysis results (donor 5). (A) UMAP visualization of clustering results for the lung cells. (B) The ratio of ACE2-expressed cells in each cell cluster. (C) The ratio of TMPRSS2-expressed cells in each cell cluster. (D) ACE2 expression level in each cell cluster on the UMPA plot. (E) TMPRSS2 expression level in each cell cluster on the UMPA plot. (F) The expression distribution of ACE2 across each cell cluster. (G) The expression distribution of TMPRSS2 across each cell cluster.

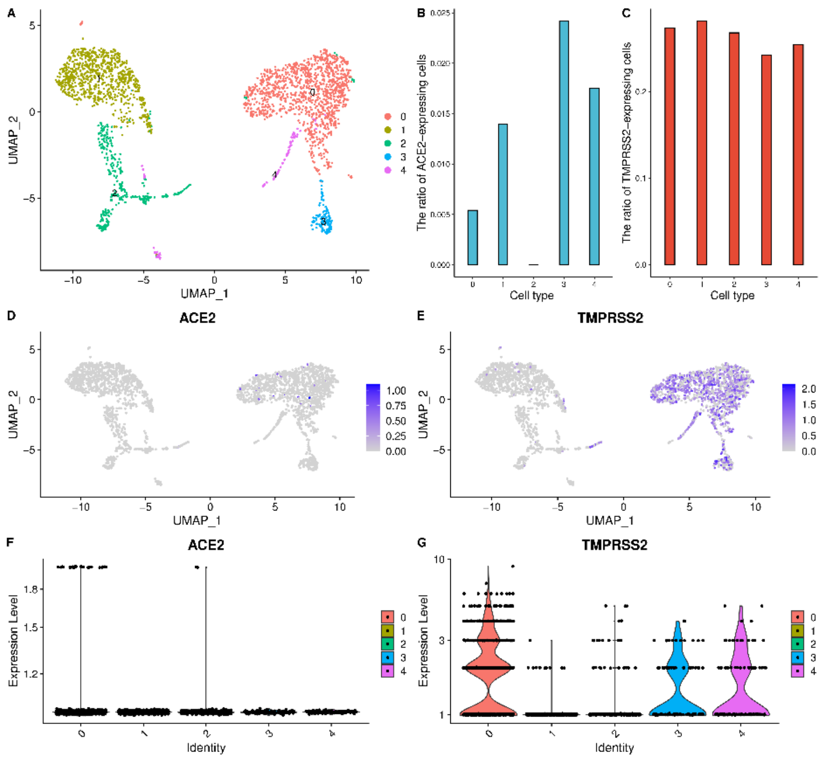


Fig. S6. The lung scRNA-seq data analysis results (donor 6). (A) UMAP visualization of clustering results for the lung cells. (B) The ratio of ACE2-expressed cells in each cell cluster. (C) The ratio of TMPRSS2-expressed cells in each cell cluster. (D) ACE2 expression level in each cell cluster on the UMPA plot. (E) TMPRSS2 expression level in each cell cluster on the UMPA plot. (F) The expression distribution of ACE2 across each cell cluster. (G) The expression distribution of TMPRSS2 across each cell cluster.

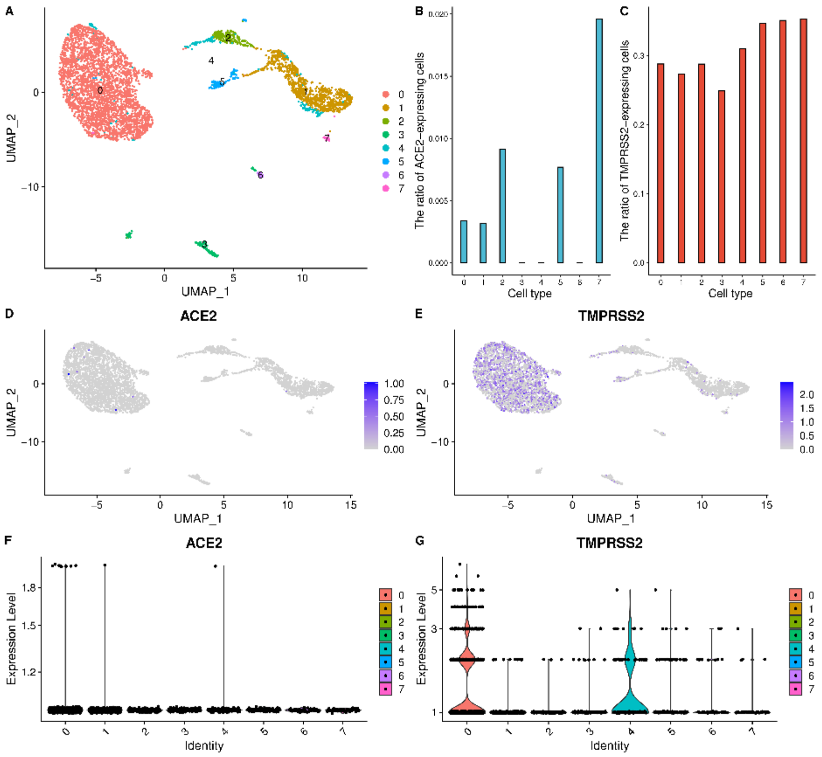


Fig. S7. The lung scRNA-seq data analysis results (donor 7). (A) UMAP visualization of clustering results for the lung cells. (B) The ratio of ACE2-expressed cells in each cell cluster. (C) The ratio of TMPRSS2-expressed cells in each cell cluster. (D) ACE2 expression level in each cell cluster on the UMPA plot. (E) TMPRSS2 expression level in each cell cluster on the UMPA plot. (F) The expression distribution of ACE2 across each cell cluster. (G) The expression distribution of TMPRSS2 across each cell cluster.

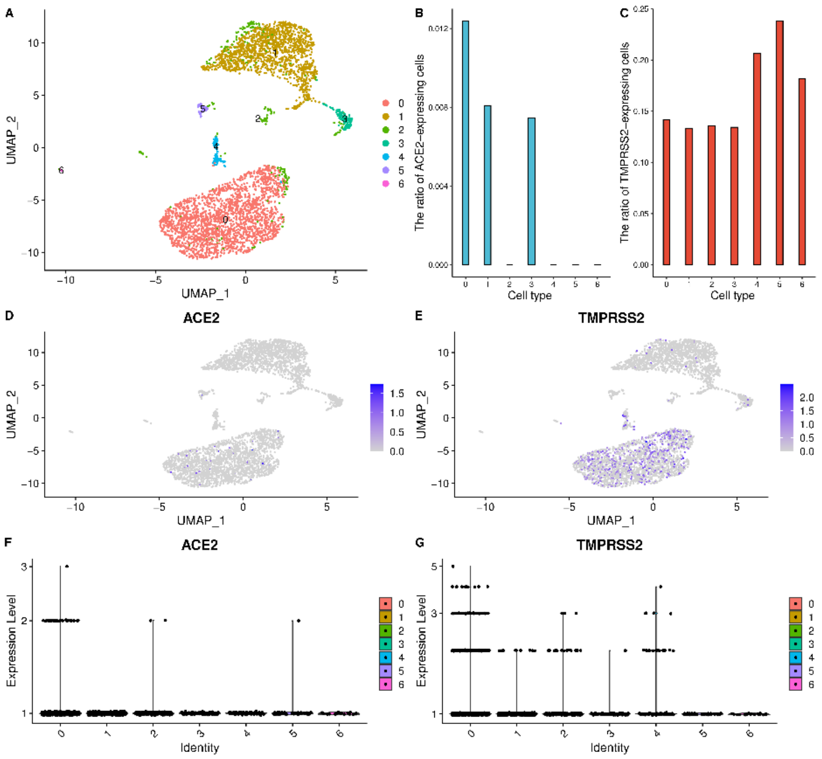


Fig. S8. The lung scRNA-seq data analysis results (donor 8). (A) UMAP visualization of clustering results for the lung cells. (B) The ratio of ACE2-expressed cells in each cell cluster. (C) The ratio of TMPRSS2-expressed cells in each cell cluster. (D) ACE2 expression level in each cell cluster on the UMPA plot. (E) TMPRSS2 expression level in each cell cluster on the UMPA plot. (F) The expression distribution of ACE2 across each cell cluster. (G) The expression distribution of TMPRSS2 across each cell cluster.

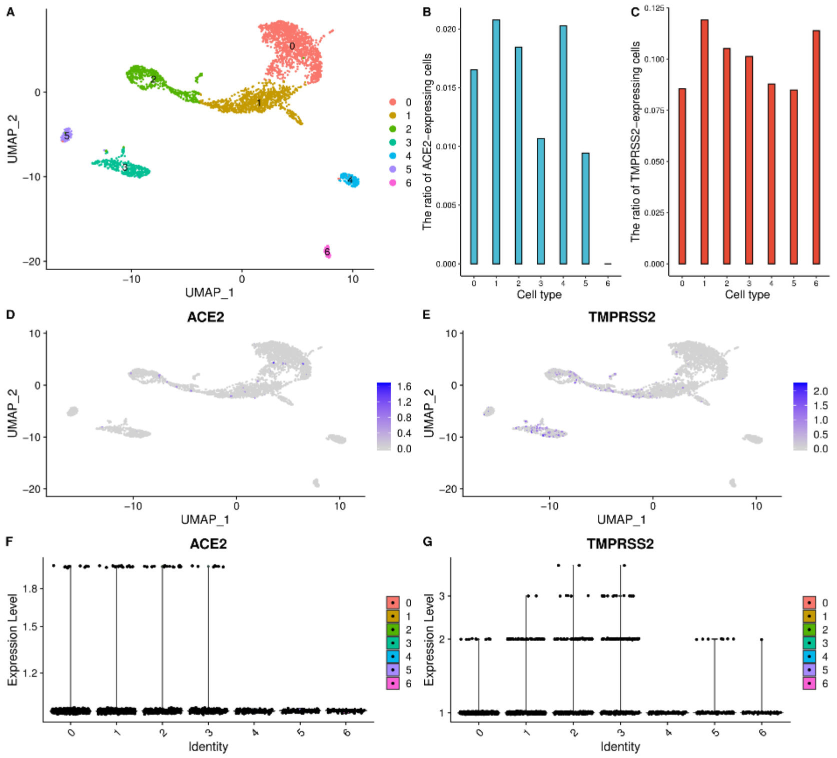


Fig. S9. High ACE2 and TMPRSS2 expression levels of the mesenchymal stromal cells, plasma cells in the nasal turbinate epithelial cells. (A) UMAP visualization of clustering results for the nasal turbinate epithelial cells. (B) The ratio of ACE2-expressed cells in each cell cluster. (C) The ratio of TMPRSS2-expressed cells in each cell cluster. (D) ACE2 expression level in each cell cluster on the UMPA plot. (E) TMPRSS2 expression level in each cell cluster on the UMPA plot. (F) The expression distribution of ACE2 across each cell cluster. (G) The expression distribution of TMPRSS2 across each cell cluster.

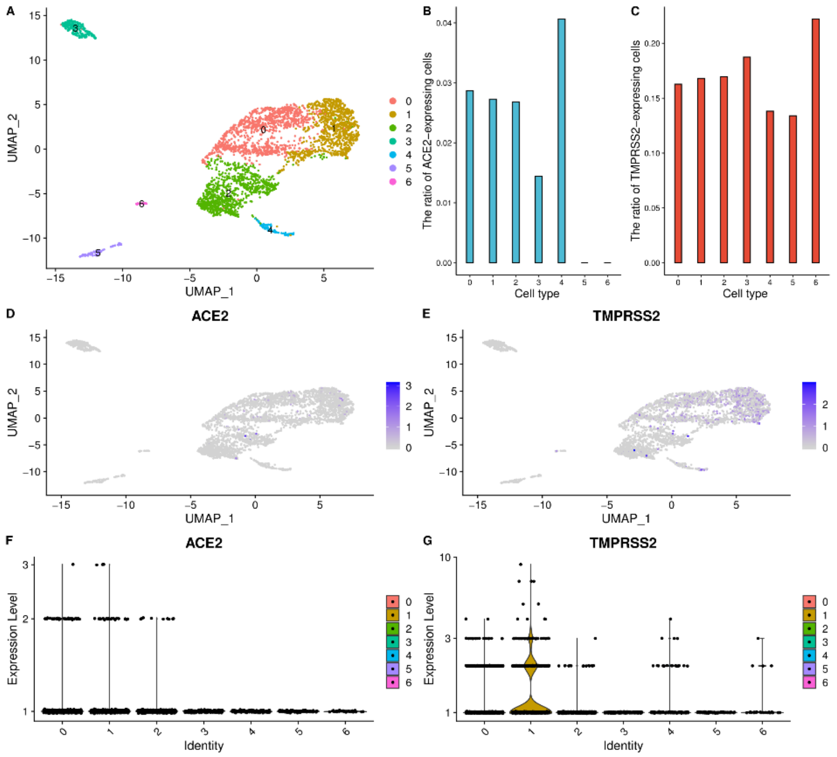


Fig. S10. High ACE2 and TMPRSS2 expression levels of the mesenchymal stromal cells, plasma cells in the nasal brushing epithelial cells. (A) UMAP visualization of clustering results for the nasal brushing epithelial cells. (B) The ratio of ACE2-expressed cells in each cell cluster. (C) The ratio of TMPRSS2-expressed cells in each cell cluster. (D) ACE2 expression level in each cell cluster on the UMPA plot. (E) TMPRSS2 expression level in each cell cluster on the UMPA plot. (F) The expression distribution of ACE2 across each cell cluster. (G) The expression distribution of TMPRSS2 across each cell cluster.

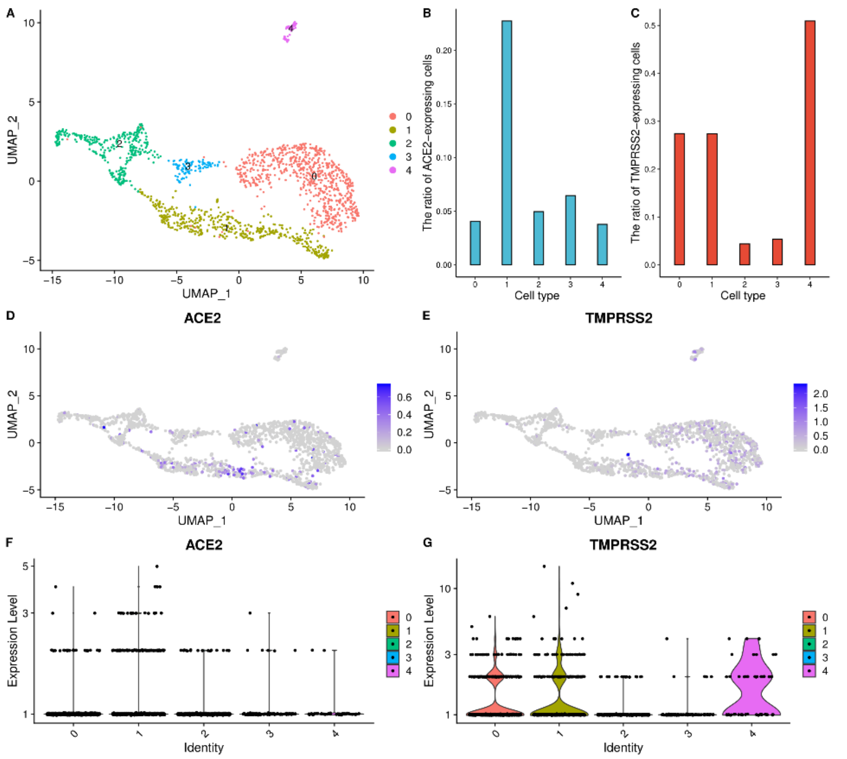


Fig. S11. High ACE2 and TMPRSS2 expression levels in the nasal airway epithelial cells. (A) UMAP visualization of clustering results for the airway epithelial cells. (B) The ratio of ACE2-expressed cells in each cell cluster. (C) The ratio of TMPRSS2-expressed cells in each cell cluster. (D) ACE2 expression level in each cell cluster on the UMPA plot. (E) TMPRSS2 expression level in each cell cluster on the UMPA plot. (F) The expression distribution of ACE2 across each cell cluster. (G) The expression distribution of TMPRSS2 across each cell cluster.

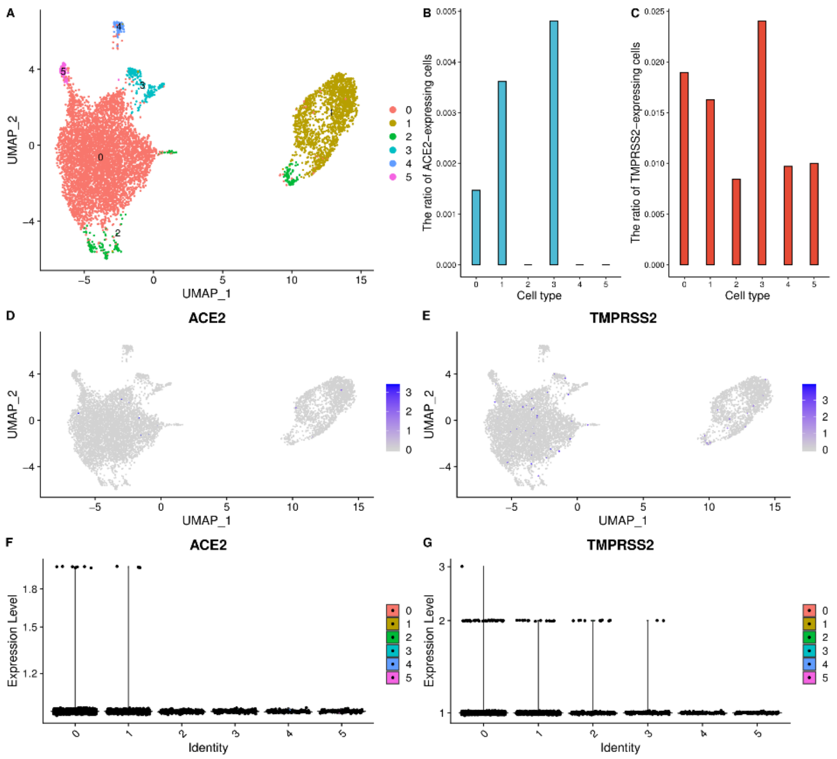


Fig. S12. The bronchus scRNA-seq data analysis results. (A) UMAP visualization of clustering results for the bronchus cells. (B) The ratio of ACE2-expressed cells in each cell cluster. (C) The ratio of TMPRSS2-expressed cells in each cell cluster. (D) ACE2 expression level in each cell cluster on the UMPA plot. (E) TMPRSS2 expression level in each cell cluster on the UMPA plot. (F) The expression distribution of ACE2 across each cell cluster. (G) The expression distribution of TMPRSS2 across each cell cluster.

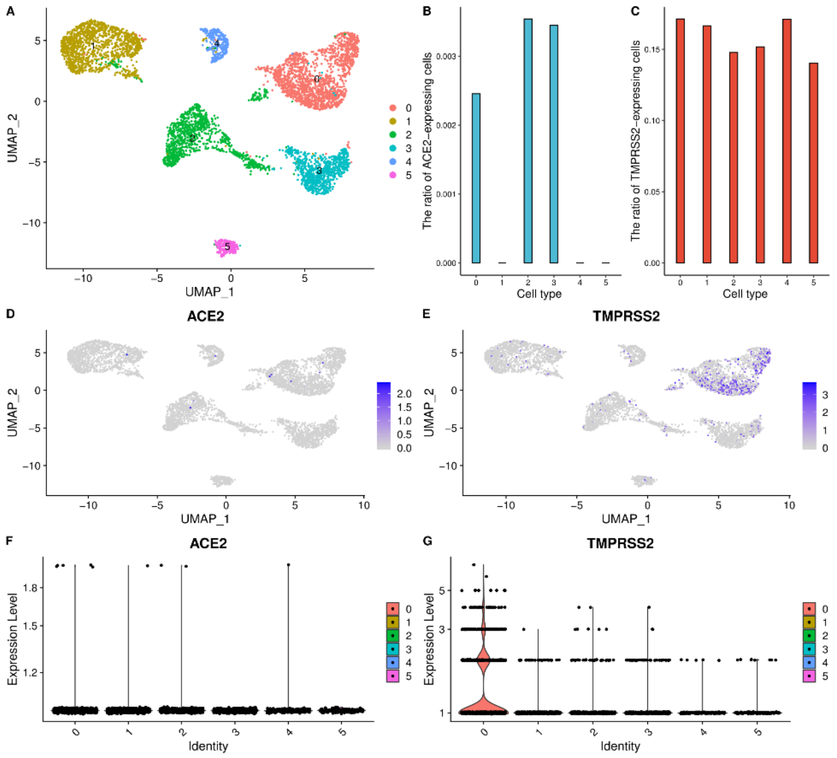


Fig. S13. The trachea scRNA-seq data analysis results. (A) UMAP visualization of clustering results for the trachea cells. (B) The ratio of ACE2-expressed cells in each cell cluster. (C) The ratio of TMPRSS2-expressed cells in each cell cluster. (D) ACE2 expression level in each cell cluster on the UMPA plot. (E) TMPRSS2 expression level in each cell cluster on the UMPA plot. (F) The expression distribution of ACE2 across each cell cluster. (G) The expression distribution of TMPRSS2 across each cell cluster.

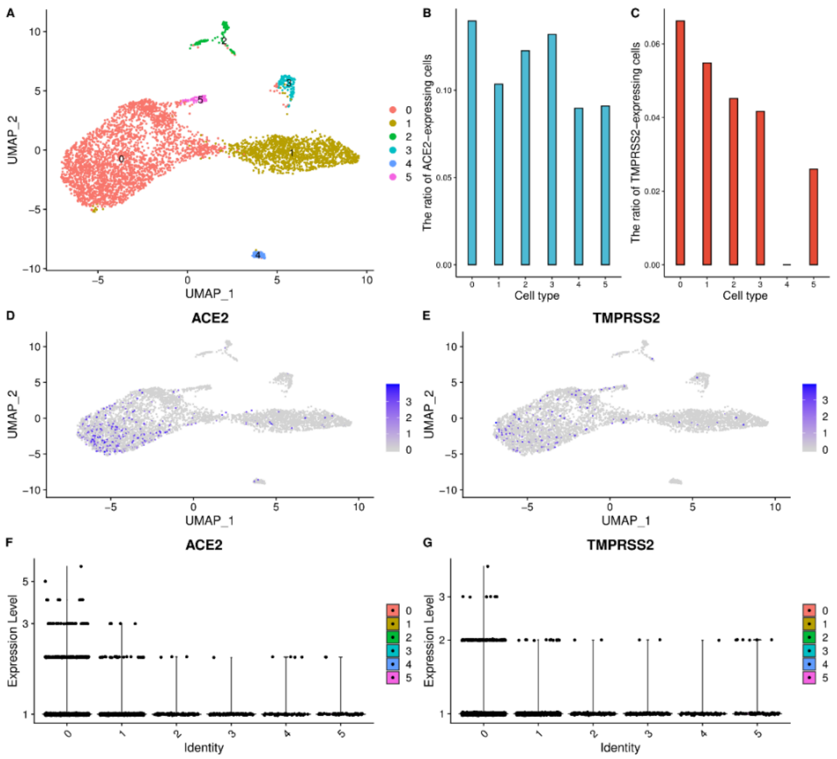


Fig. S14. High ACE2 and TMPRSS2 expression level of enterocyte progenitor cells and goblet cells in the jejunum. (A) UMAP visualization of clustering results for jejunum cells. (B) The ratio of ACE2-expressed cells in each cell cluster. (C) The ratio of TMPRSS2-expressed cells in each cell cluster. (D) ACE2 expression level in each cell cluster on the UMPA plot. (E) TMPRSS2 expression level in each cell cluster on the UMPA plot. (F) The expression distribution of ACE2 across each cell cluster. (G) The expression distribution of TMPRSS2 across each cell cluster.

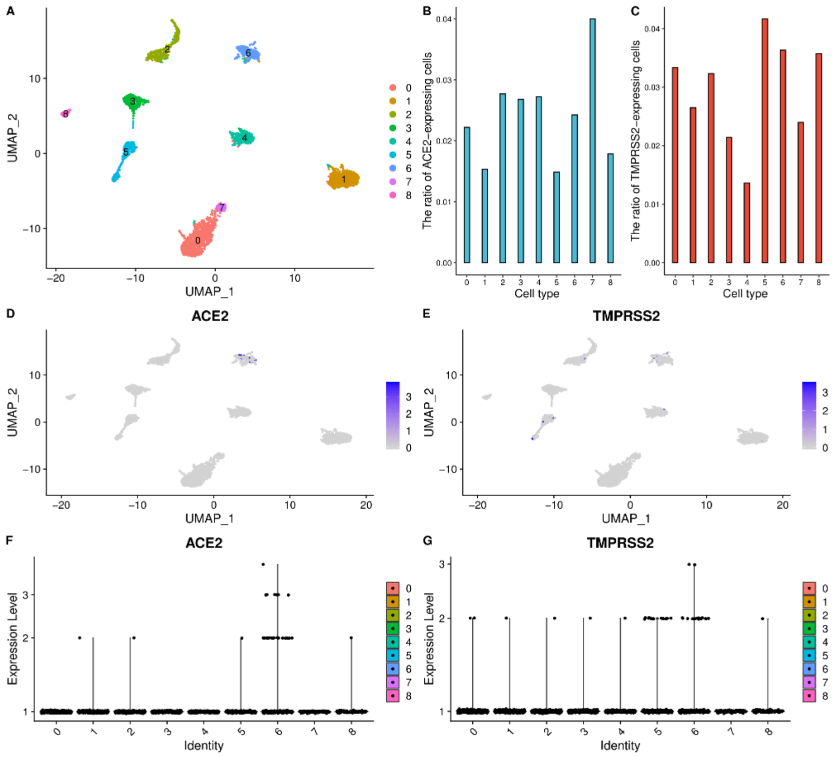


Fig. S15. High ACE2 and TMPRSS2 expression level of the intestinal epithelial stem cells and enterocyte progenitor cells in the ileum. (A) UMAP visualization of clustering results for ileum cells. (B) The ratio of ACE2-expressed cells in each cell cluster. (C) The ratio of TMPRSS2-expressed cells in each cell cluster. (D) ACE2 expression level in each cell cluster on the UMPA plot. (E) TMPRSS2 expression level in each cell cluster on the UMPA plot. (F) The expression distribution of ACE2 across each cell cluster. (G) The expression distribution of TMPRSS2 across each cell cluster.

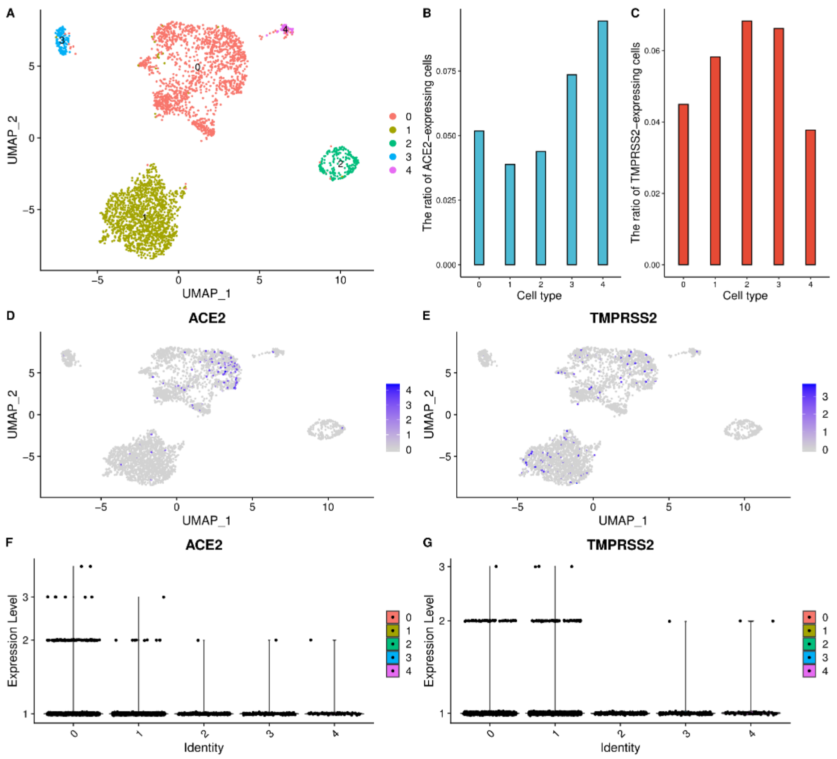


Fig. S16. High ACE2 and TMPRSS2 expression level of the intestinal LGR5+ stem cells, epithelial stem cells, enterocyte progenitor cells, tuft progenitor cells, and enteroendocrine cells in the duodenum. (A) UMAP visualization of clustering results for duodenum cells. (B) The ratio of ACE2-expressed cells in each cell cluster. (C) The ratio of TMPRSS2-expressed cells in each cell cluster. (D) ACE2 expression level in each cell cluster on the UMPA plot. (E) TMPRSS2 expression level in each cell cluster on the UMPA plot. (F) The expression distribution of ACE2 across each cell cluster. (G) The expression distribution of TMPRSS2 across each cell cluster.

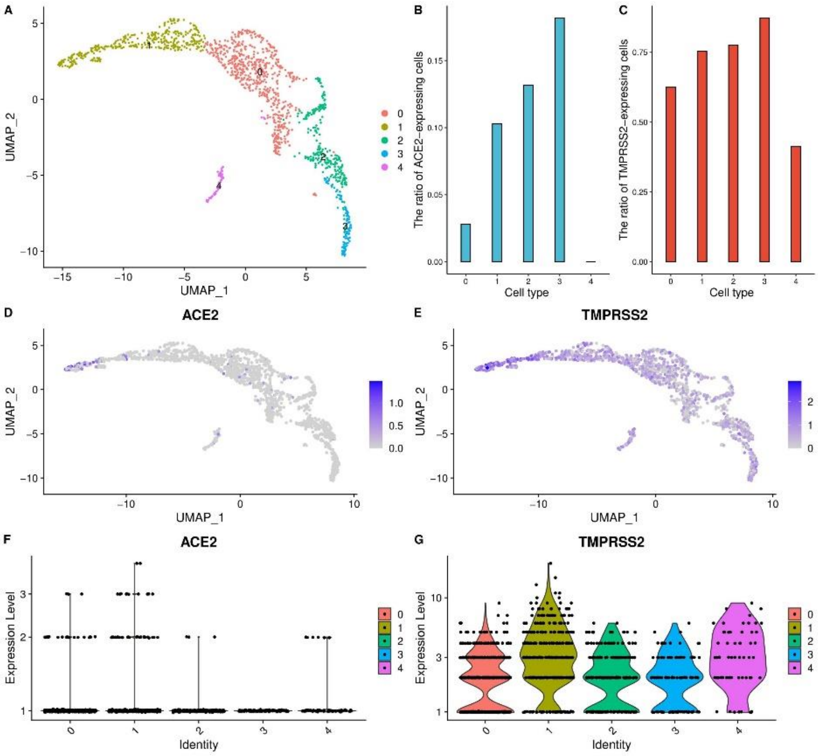


Fig. S17. High ACE2 and TMPRSS2 expression level of goblet progenitor cells, MKI67+ progenitor cells, enterocytes, and goblet cells in the rectum. (A) UMAP visualization of clustering results for rectum cells. (B) The ratio of ACE2-expressed cells in each cell cluster. (C) The ratio of TMPRSS2-expressed cells in each cell cluster. (D) ACE2 expression level in each cell cluster on the UMPA plot. (E) TMPRSS2 expression level in each cell cluster on the UMPA plot. (F) The expression distribution of ACE2 across each cell cluster. (G) The expression distribution of TMPRSS2 across each cell cluster.

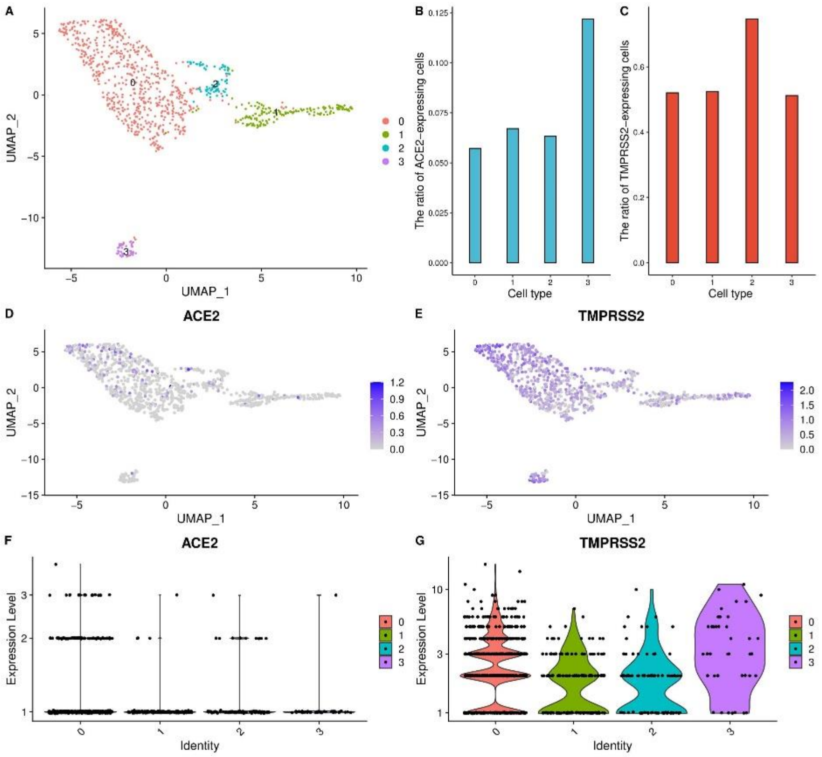


Fig. S18. High ACE2 and TMPRSS2 expression level of the enterocytes and goblet cells in the colon. (A) UMAP visualization of clustering results for colon cells. (B) The ratio of ACE2-expressed cells in each cell cluster. (C) The ratio of TMPRSS2-expressed cells in each cell cluster. (D) ACE2 expression level in each cell cluster on the UMPA plot. (E) TMPRSS2 expression level in each cell cluster on the UMPA plot. (F) The expression distribution of ACE2 across each cell cluster. (G) The expression distribution of TMPRSS2 across each cell cluster.

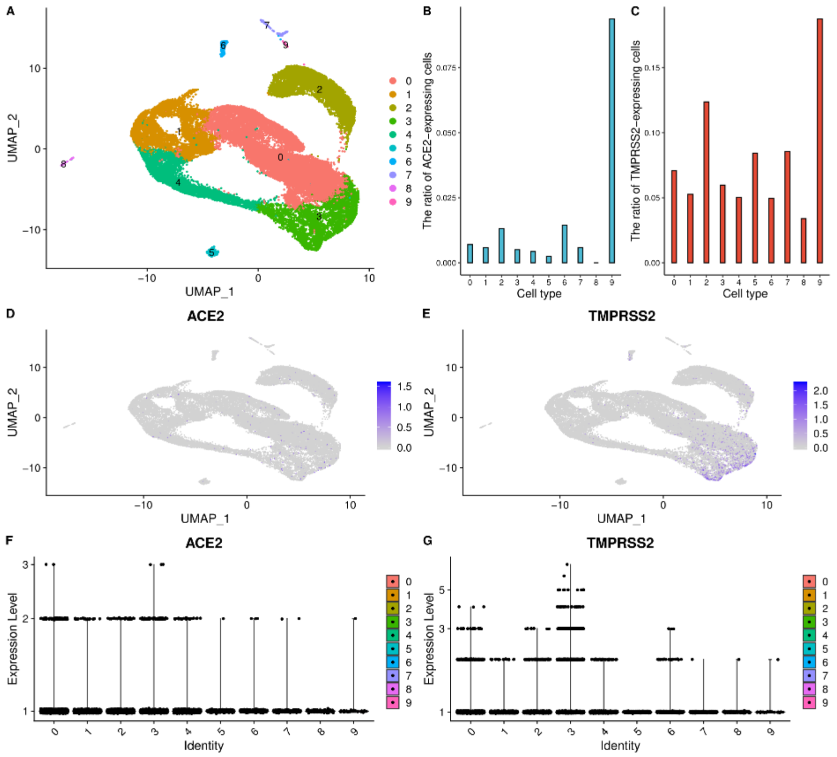


Fig. S19. High ACE2 and TMPRSS2 expression level of the secretory progenitor cells in the esophagus. (A) UMAP visualization of clustering results for esophagus cells. (B) The ratio of ACE2-expressed cells in each cell cluster. (C) The ratio of TMPRSS2-expressed cells in each cell cluster. (D) ACE2 expression level in each cell cluster on the UMPA plot. (E) TMPRSS2 expression level in each cell cluster on the UMPA plot. (F) The expression distribution of ACE2 across each cell cluster. (G) The expression distribution of TMPRSS2 across each cell cluster.

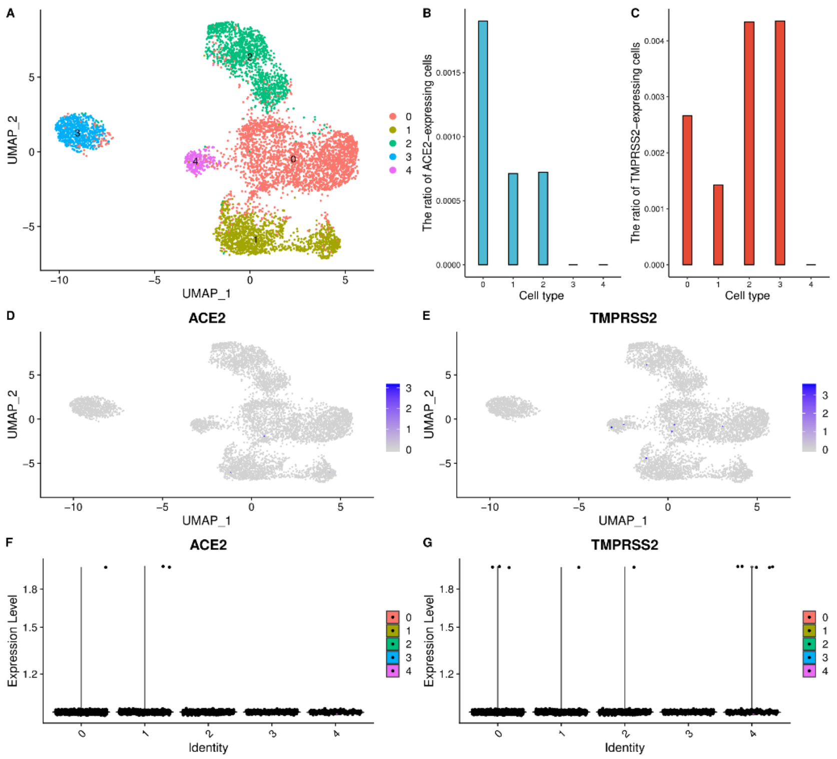


Fig. S20. The liver scRNA-seq data analysis results. (A) UMAP visualization of clustering results for liver cells. (B) The ratio of ACE2-expressed cells in each cell cluster. (C) The ratio of TMPRSS2-expressed cells in each cell cluster. (D) ACE2 expression level in each cell cluster on the UMPA plot. (E) TMPRSS2 expression level in each cell cluster on the UMPA plot. (F) The expression distribution of ACE2 across each cell cluster. (G) The expression distribution of TMPRSS2 across each cell cluster.

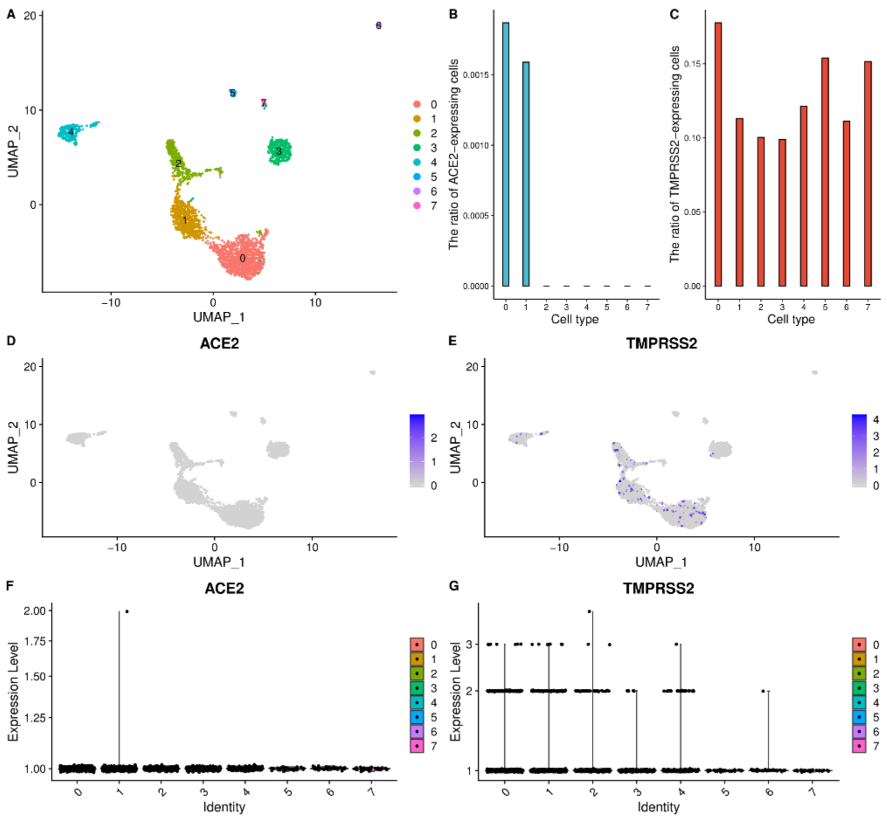


Fig. S21. The stomach scRNA-seq data analysis results. (A) UMAP visualization of clustering results for stomach cells. (B) The ratio of ACE2-expressed cells in each cell cluster. (C) The ratio of TMPRSS2-expressed cells in each cell cluster. (D) ACE2 expression level in each cell cluster on the UMPA plot. (E) TMPRSS2 expression level in each cell cluster on the UMPA plot. (F) The expression distribution of ACE2 across each cell cluster. (G) The expression distribution of TMPRSS2 across each cell cluster.

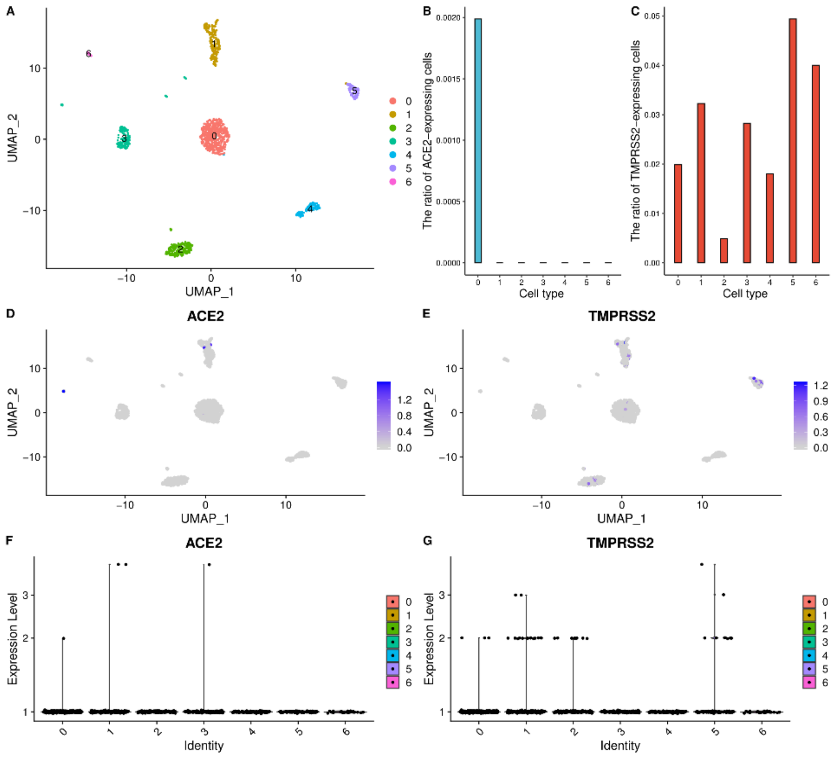


Fig. S22. The pancreatic islets scRNA-seq data analysis results. (A) UMAP visualization of clustering results for pancreatic islets cells. (B) The ratio of ACE2-expressed cells in each cell cluster. (C) The ratio of TMPRSS2-expressed cells in each cell cluster. (D) ACE2 expression level in each cell cluster on the UMPA plot. (E) TMPRSS2 expression level in each cell cluster on the UMPA plot. (F) The expression distribution of ACE2 across each cell cluster. (G) The expression distribution of TMPRSS2 across each cell cluster.

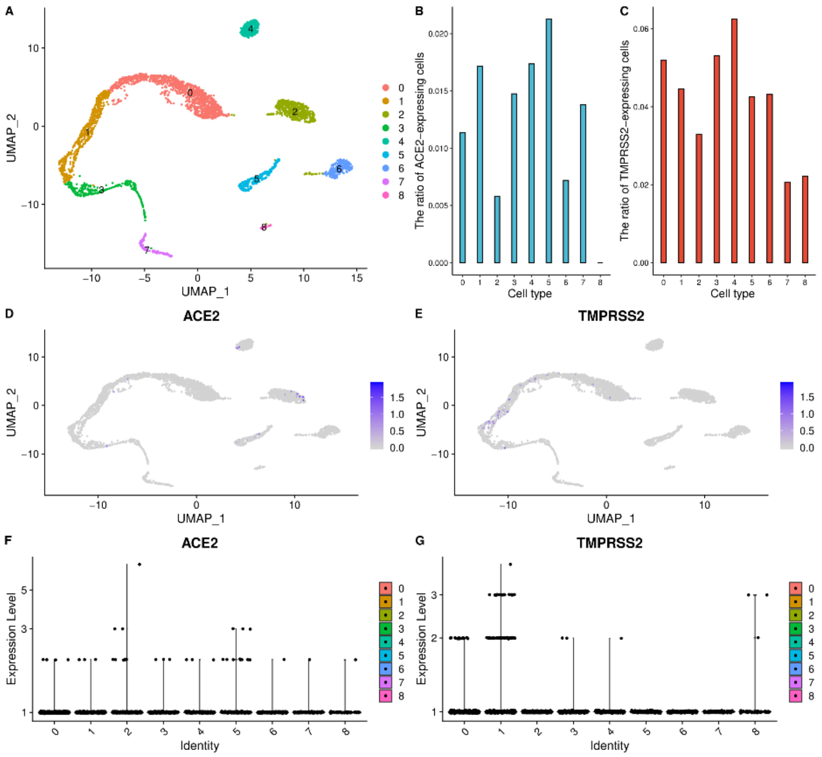

Fig. S23. High ACE2 and TMPRSS2 expression level of spermatogonium, peritubular myoid cells, testis somatic cells, and spermatogonial stem cells in the testis. (A) UMAP visualization of clustering results for testis cells. (B) The ratio of ACE2-expressed cells in each cell cluster. (C) The ratio of TMPRSS2-expressed cells in each cell cluster. (D) ACE2 expression level in each cell cluster on the UMPA plot. (E) TMPRSS2 expression level in each cell cluster on the UMPA plot. (F) The expression distribution of ACE2 across each cell cluster. (G) The expression distribution of TMPRSS2 across each cell cluster.

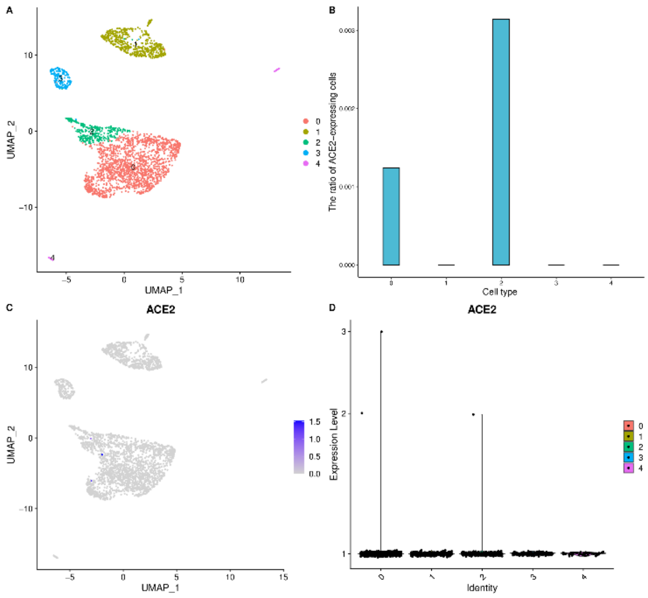


Fig. S24. The ovary scRNA-seq data analysis results. (A) UMAP visualization of clustering results for ovary cells. (B) The ratio of ACE2-expressed cells in each cell cluster. (C) ACE2 expression level in each cell cluster on the UMPA plot. (D) The expression distribution of ACE2 across each cell cluster.

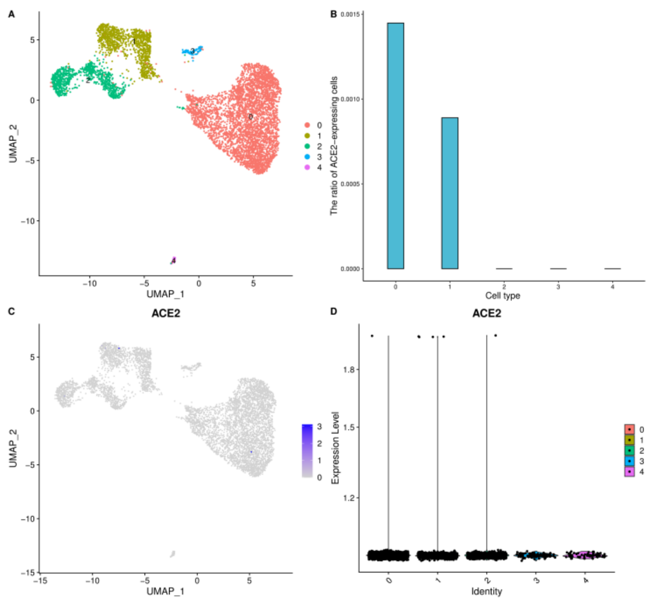


Fig. S25. The uterus scRNA-seq data analysis results. (A) UMAP visualization of clustering results for uterus cells. (B) The ratio of ACE2-expressed cells in each cell cluster. (C) ACE2 expression level in each cell cluster on the UMPA plot. (D) The expression distribution of ACE2 across each cell cluster.

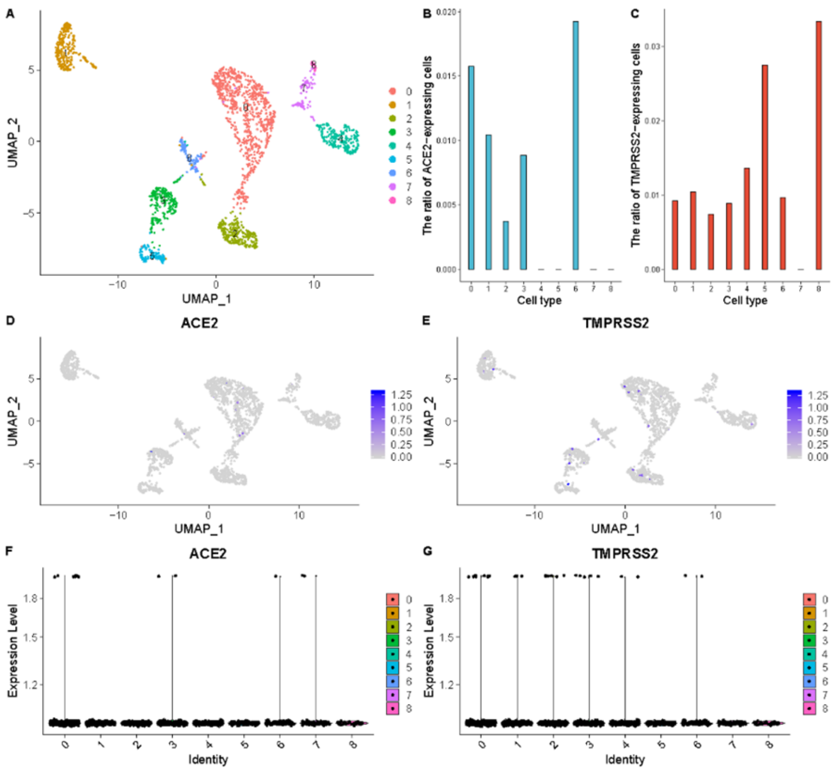


Fig. S26. High ACE2 and TMPRSS2 expression level of oligodendrocyte precursor cells and astrocytes in the substantia nigra and cortex. (A) UMAP visualization of clustering results for the substantia nigra and cortex cells. (B) The ratio of ACE2-expressed cells in each cell cluster. (C) The ratio of TMPRSS2-expressed cells in each cell cluster. (D) ACE2 expression level in each cell cluster on the UMPA plot. (E) TMPRSS2 expression level in each cell cluster on the UMPA plot. (F) The expression distribution of ACE2 across each cell cluster. (G) The expression distribution of TMPRSS2 across each cell cluster.

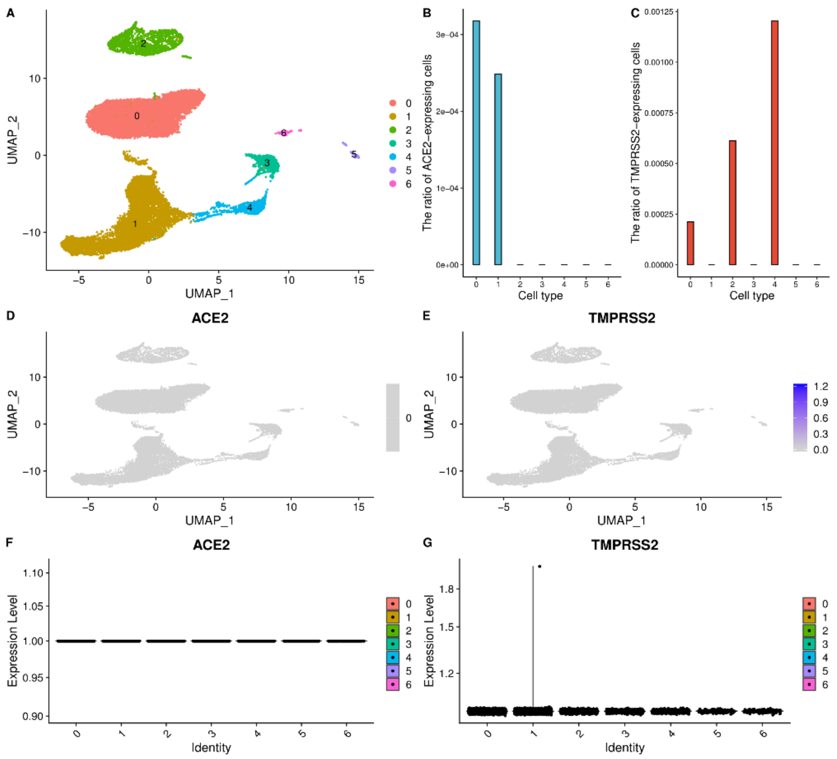

Fig. S27. The hippocampus scRNA-seq data analysis results. (A) UMAP visualization of clustering results for hippocampus cells. (B) The ratio of ACE2-expressed cells in each cell cluster. (C) The ratio of TMPRSS2-expressed cells in each cell cluster. (D) ACE2 expression level in each cell cluster on the UMPA plot. (E) TMPRSS2 expression level in each cell cluster on the UMPA plot. (F) The expression distribution of ACE2 across each cell cluster. (G) The expression distribution of TMPRSS2 across each cell cluster.

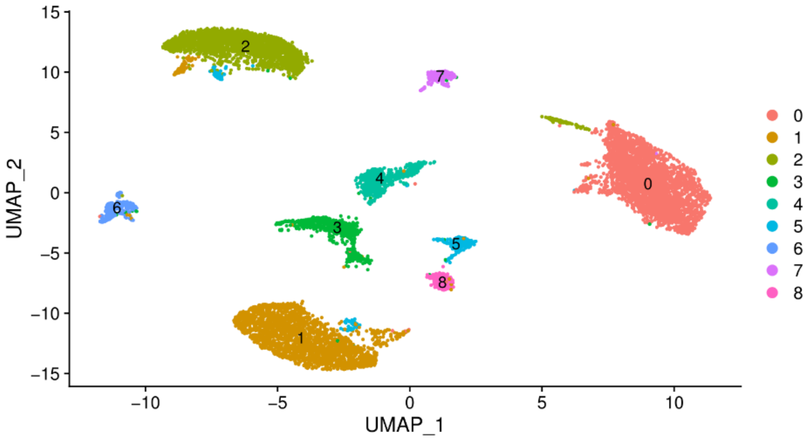


Fig. S28. UMAP visualization of clustering results for the cerebellum cells.

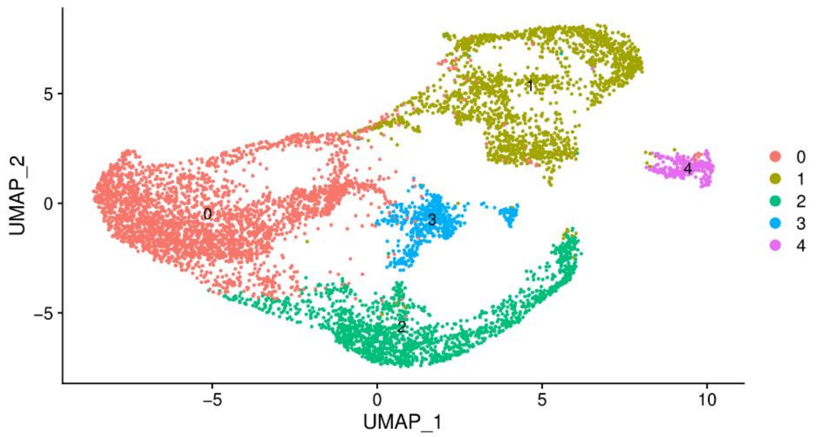


Fig. S29. UMAP visualization of clustering results for the spinal cord cells.

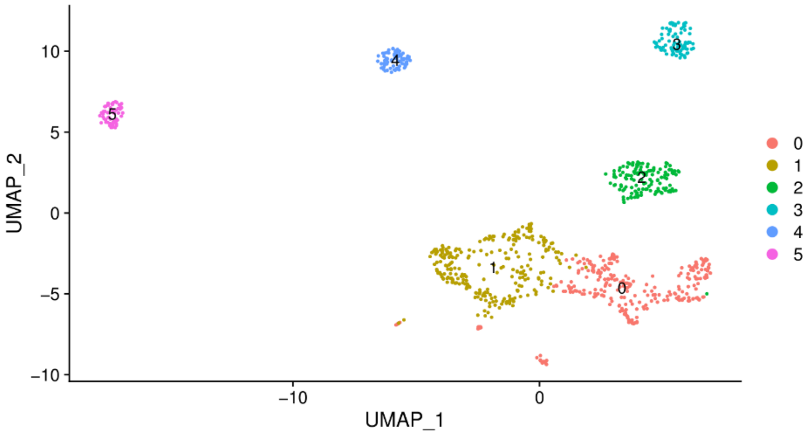


Fig. S30. UMAP visualization of clustering results for the neuronal epithelium cells.

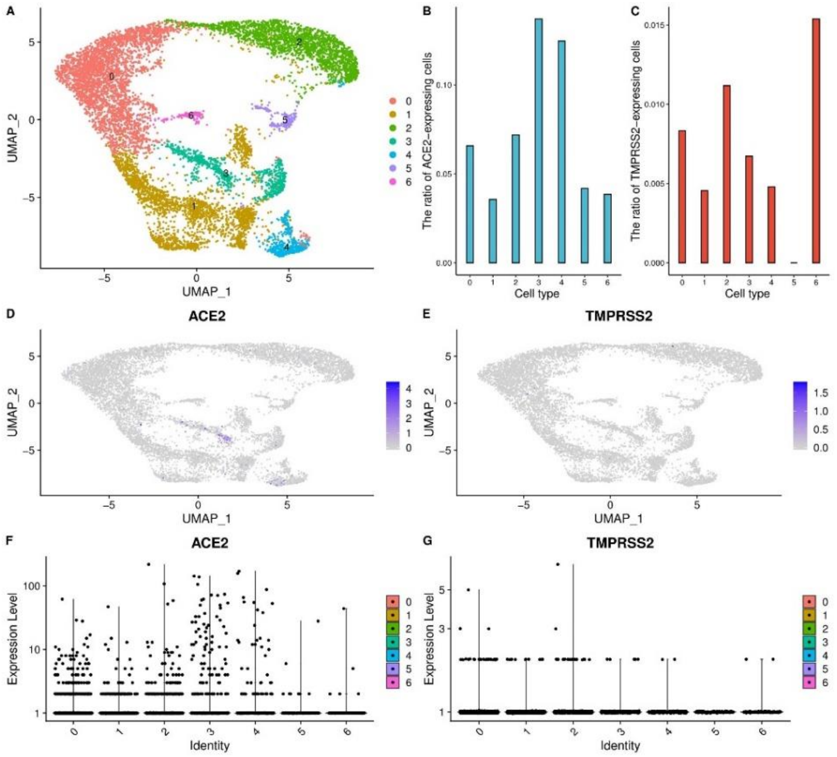


Fig. S31. High ACE2 and TMPRSS2 expression level of the cardiomyocytes and cardiovascular progenitor cells in the heart. (A) UMAP visualization of clustering results for the heart cells. (B) The ratio of ACE2-expressed cells in each cell cluster. (C) The ratio of TMPRSS2-expressed cells in each cell cluster. (D) ACE2 expression level in each cell cluster on the UMPA plot. (E) TMPRSS2 expression level in each cell cluster on the UMPA plot. (F) The expression distribution of ACE2 across each cell cluster. (G) The expression distribution of TMPRSS2 across each cell cluster.

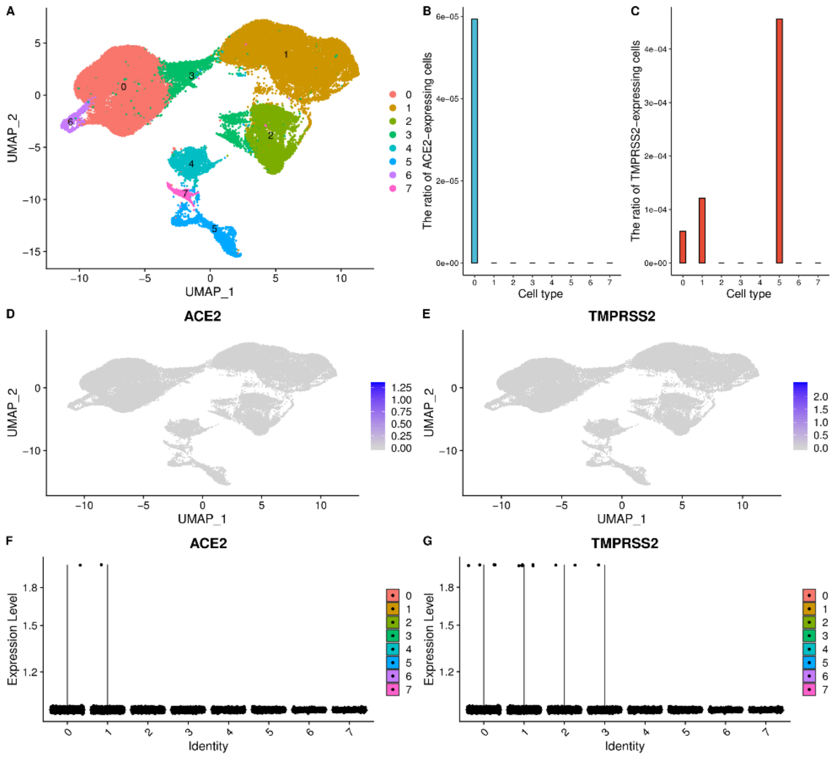


Fig. S32. The spleen scRNA-seq data analysis results. (A) UMAP visualization of clustering results for the spleen cells. (B) The ratio of ACE2-expressed cells in each cell cluster. (C) The ratio of TMPRSS2-expressed cells in each cell cluster. (D) ACE2 expression level in each cell cluster on the UMPA plot. (E) TMPRSS2 expression level in each cell cluster on the UMPA plot. (F) The expression distribution of ACE2 across each cell cluster. (G) The expression distribution of TMPRSS2 across each cell cluster.

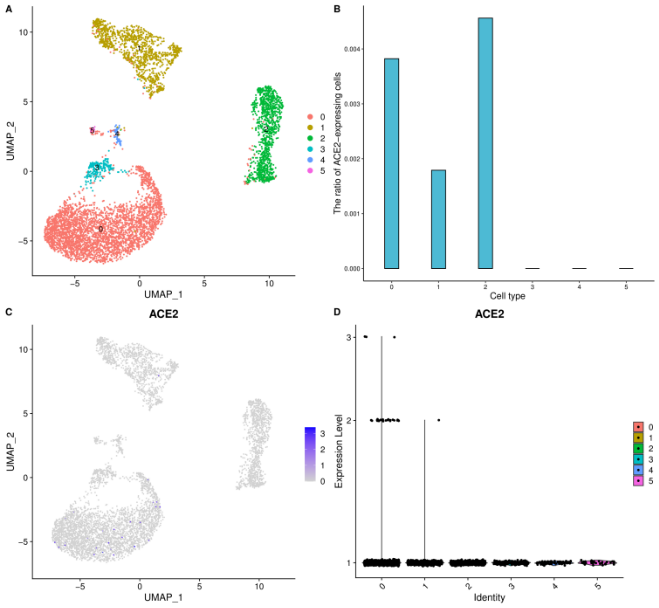


Fig. S33. The artery scRNA-seq data analysis results. (A) UMAP visualization of clustering results for the spleen cells. (B) The ratio of ACE2-expressed cells in each cell cluster. (C) ACE2 expression level in each cell cluster on the UMPA plot. (D) The expression distribution of ACE2 across each cell cluster.

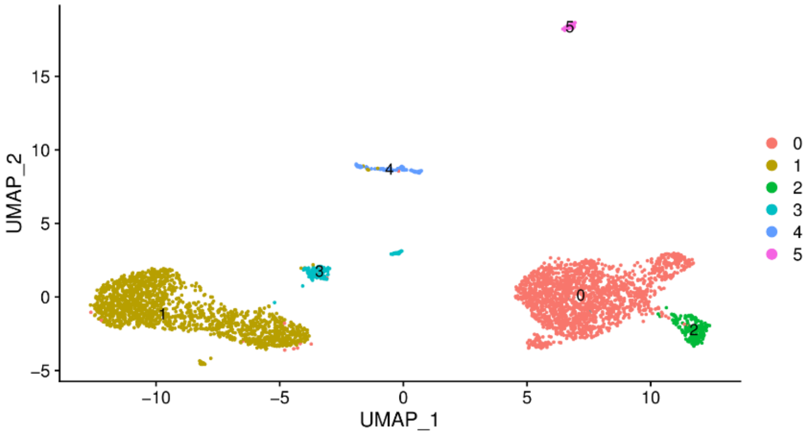


Fig. S34. UMAP visualization of clustering results for the peripheral blood cells.

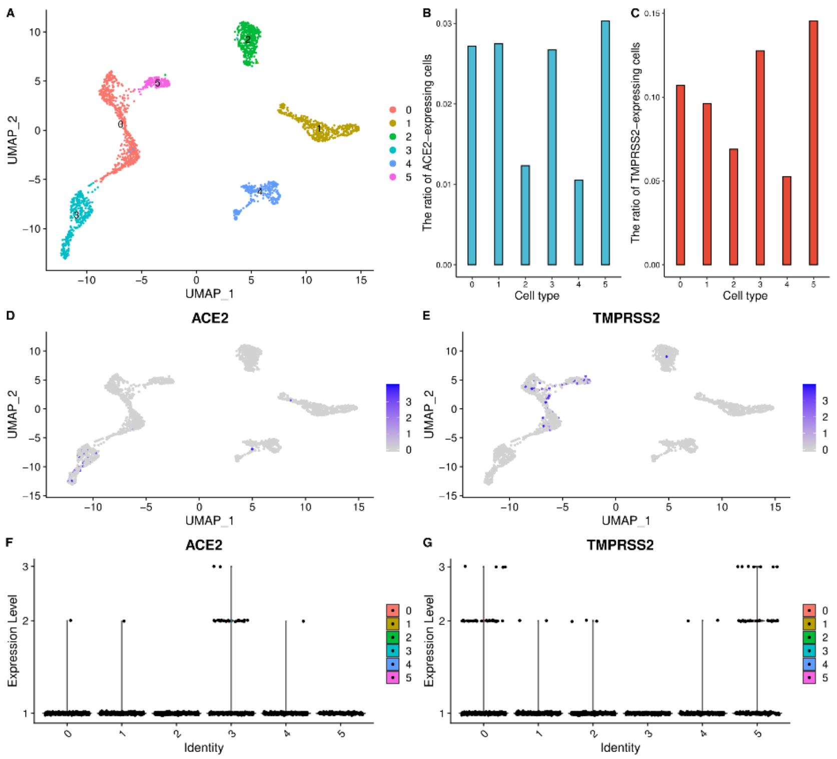


Fig. S35. High ACE2 and TMPRSS2 expression level of the nephron epithelial cells, epithelial cells, endothelial cells, and mesangial cells in the kidney. (A) UMAP visualization of clustering results for the kidney cells. (B) The ratio of ACE2-expressed cells in each cell cluster. (C) The ratio of TMPRSS2-expressed cells in each cell cluster. (D) ACE2 expression level in each cell cluster on the UMPA plot. (E) TMPRSS2 expression level in each cell cluster on the UMPA plot. (F) The expression distribution of ACE2 across each cell cluster. (G) The expression distribution of TMPRSS2 across each cell cluster.

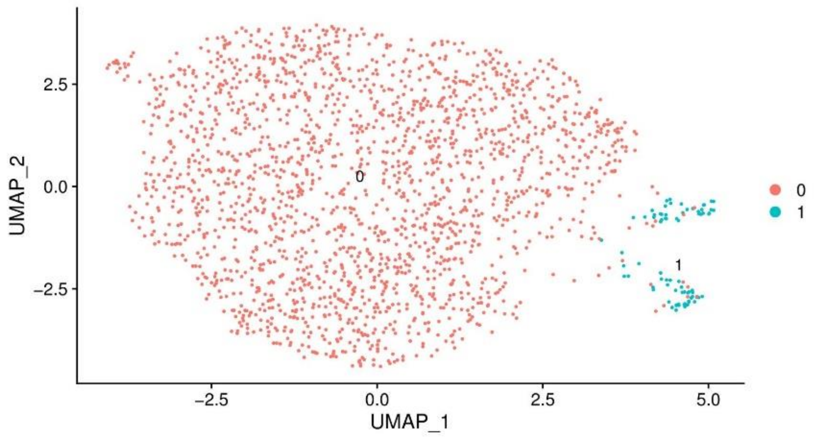


Fig. S36. UMAP visualization of clustering results for the ureter cells.

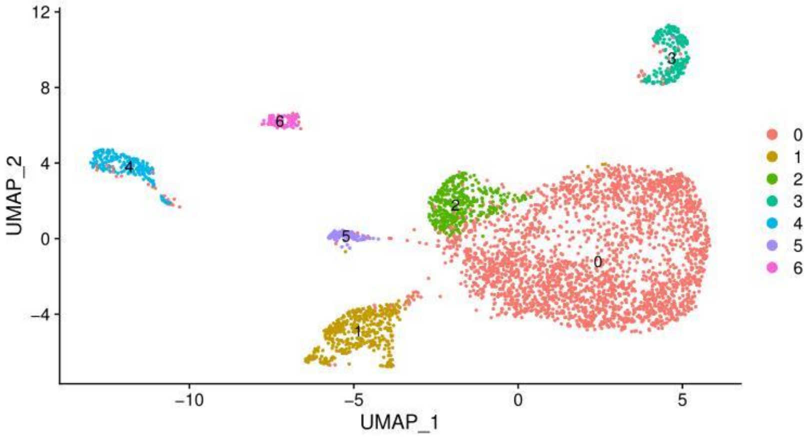


Fig. S37. UMAP visualization of clustering results for the prostate cells.

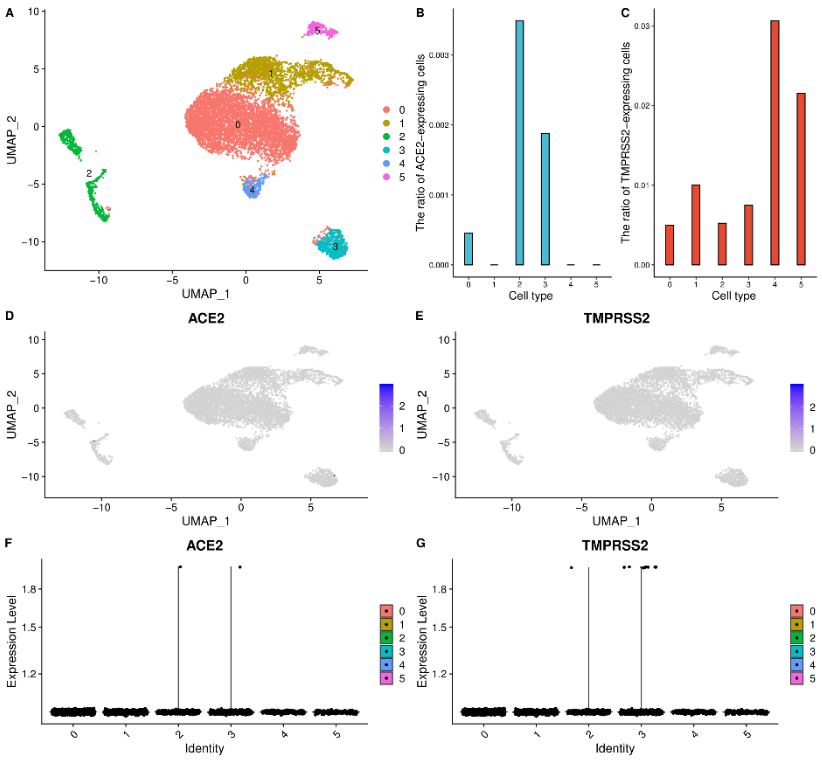


Fig. S38. The thyroid gland scRNA-seq data analysis results. (A) UMAP visualization of clustering results for the thyroid gland cells. (B) The ratio of ACE2-expressed cells in each cell cluster. (C) The ratio of TMPRSS2-expressed cells in each cell cluster. (D) ACE2 expression level in each cell cluster on the UMPA plot. (E) TMPRSS2 expression level in each cell cluster on the UMPA plot. (F) The expression distribution of ACE2 across each cell cluster. (G) The expression distribution of TMPRSS2 across each cell cluster.

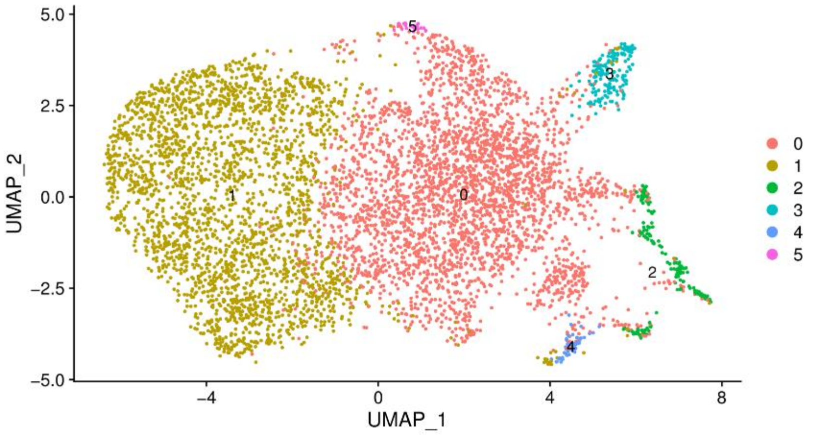


Fig. S39. UMAP visualization of clustering results for the thymus gland cells.

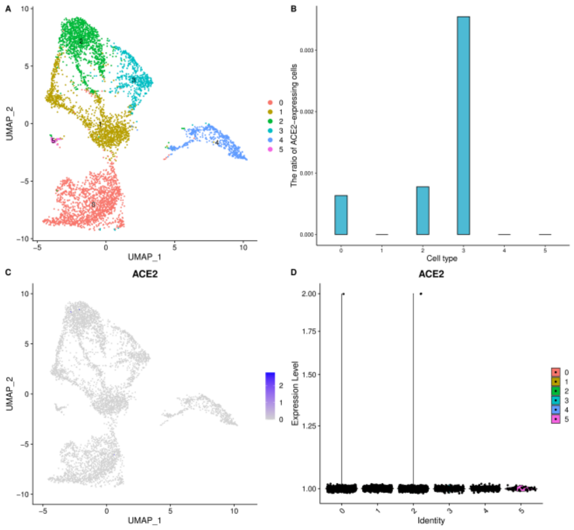


Fig. S40. The muscle scRNA-seq data analysis results. (A) UMAP visualization of clustering results for the muscle cells. (B) The ratio of ACE2-expressed cells in each cell cluster. (C) ACE2 expression level in each cell cluster on the UMPA plot. (D) The expression distribution of ACE2 across each cell cluster.

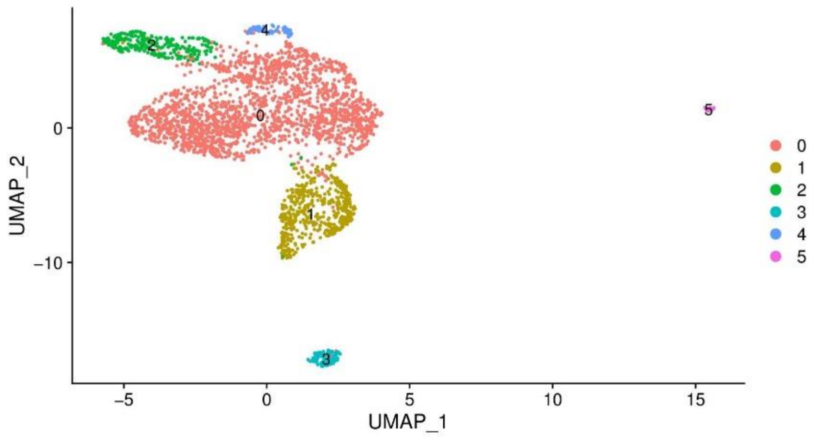


Fig.. S41. UMAP visualization of clustering results for the lymph nodes cells.

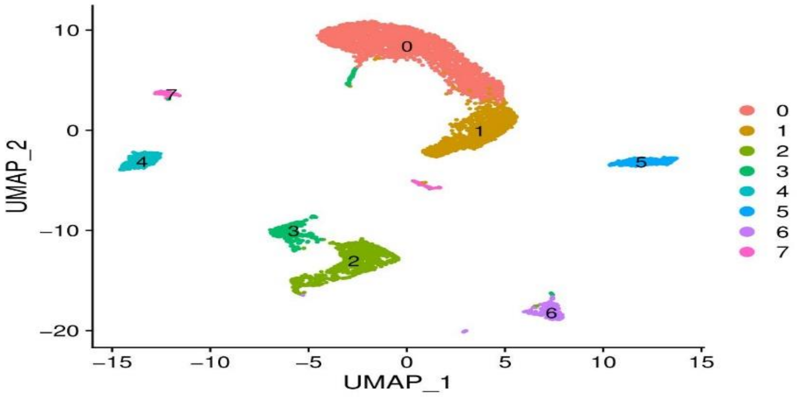


Fig. S42. UMAP visualization of clustering results for the tonsil dendritic cells.

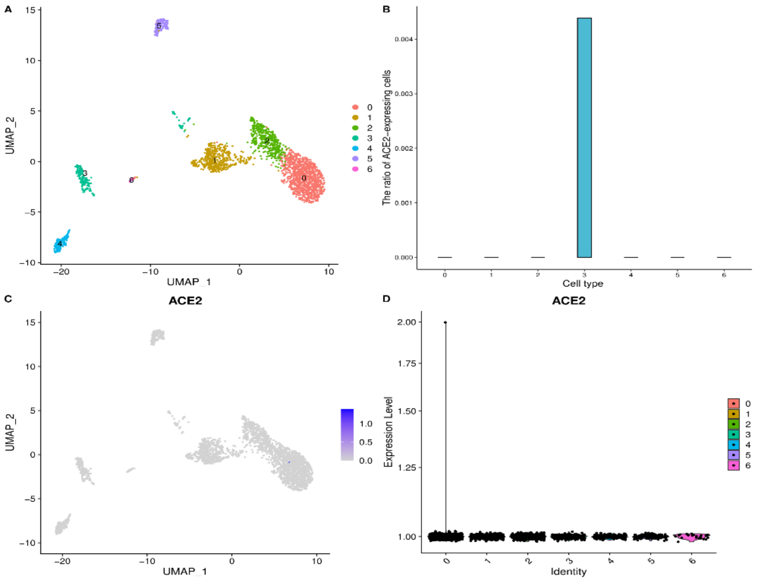


Fig. S43. The bone marrow scRNA-seq data analysis results. (A) UMAP visualization of clustering results for the bone marrow cells. (B) The ratio of ACE2-expressed cells in each cell cluster. (C) ACE2 expression level in each cell cluster on the UMPA plot. (D) The expression distribution of ACE2 across each cell cluster.
